# Accelerated replicative senescence of ataxia‐telangiectasia skin fibroblasts is retained at physiologic oxygen levels, with unique and common transcriptional patterns

**DOI:** 10.1111/acel.13869

**Published:** 2023-05-30

**Authors:** Majd Haj, Amit Levon, Yann Frey, Noa Hourvitz, Judith Campisi, Yehuda Tzfati, Ran Elkon, Yael Ziv, Yosef Shiloh

**Affiliations:** ^1^ Department of Human Molecular Genetics and Biochemistry Tel Aviv University School of Medicine Tel Aviv Israel; ^2^ The Alexander Silberman Institute of Life Sciences The Hebrew University of Jerusalem Jerusalem Israel; ^3^ Buck Institute for Research on Aging Novato California USA

**Keywords:** ataxia‐telangiectasia, cell senescence, oxygen concentration, transcriptome

## Abstract

The genetic disorder, ataxia‐telangiectasia (A‐T), is caused by loss of the homeostatic protein kinase, ATM, and combines genome instability, tissue degeneration, cancer predisposition, and premature aging. Primary fibroblasts from A‐T patients exhibit premature senescence when grown at ambient oxygen concentration (21%). Here, we show that reducing oxygen concentration to a physiological level range (3%) dramatically extends the proliferative lifespan of human A‐T skin fibroblasts. However, they still undergo senescence earlier than control cells grown under the same conditions and exhibit high genome instability. Comparative RNA‐seq analysis of A‐T and control fibroblasts cultured at 3% oxygen followed by cluster analysis of differentially expressed genes and functional enrichment analysis, revealed distinct transcriptional dynamics in A‐T fibroblasts senescing in physiological oxygen concentration. While some transcriptional patterns were similar to those observed during replicative senescence of control cells, others were unique to the senescing A‐T cells. We observed in them a robust activation of interferon‐stimulated genes, with undetected expression the interferon genes themselves. This finding suggests an activation of a non‐canonical cGAS‐STING‐mediated pathway, which presumably responds to cytosolic DNA emanating from extranuclear micronuclei detected in these cells. Senescing A‐T fibroblasts also exhibited a marked, intriguely complex alteration in the expression of genes associated with extracellular matrix (ECM) remodeling. Notably, many of the induced ECM genes encode senescence‐associated secretory phenotype (SASP) factors known for their paracrine pro‐fibrotic effects. Our data provide a molecular dimension to the segmental premature aging observed in A‐T patients and its associated symptoms, which develop as the patients advance in age.

## Graphical abstract

Primary fibroblasts derived from patients with the genetic disorder, ataxia‐telangiectasia (A‐T) undergo premature senescence under ambient oxygen concentration. We found that low (3%) oxygen markedly extends the life span of control and A‐T fibroblasts, but the latter still senesce prematurely. Transcriptome analysis showed robust activation of interferon‐stimulated genes (ISGs) in A‐T cells senescing under 3% oxygen, but expression of interferon genes was not detected, suggesting a non‐canonical pathway of ISG activation. Senescing A‐T fibroblasts also demonstrated a unique senescence‐associated secretory phenotype, along with deregulation of extracellular matrix (ECM) genes.

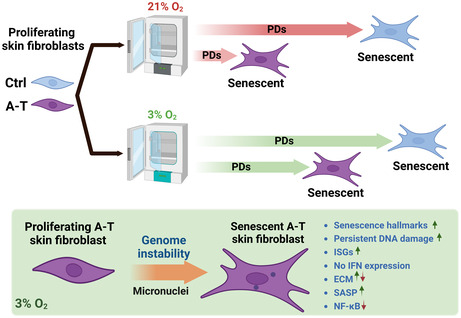

**Abbreviations**
A‐T: ataxia‐telangiectasia
ATM: ataxia‐telangiectasia, mutated
cGAS: cyclic GMP‐AMP synthase
CPD: cumulative population doubling
DDR: DNA damage response
DEG: differentially expressed gene
EMT: epithelial‐to‐mesenchymal transition
GO: gene ontology
GSEA: Gene set enrichment analysis
HGPS: Hutchinson‐Gilford progeria syndrome
IFN: interferon
ISG: interferon‐stimulated gene
MTL: mean TRF length
PCA: principal component analysis
PD: population doubling
qPCR: real‐time PCR
SA‐β‐Gal: senescence‐associated β‐galactosidase
SASP: senescence‐associated secretory phenotype
STAT: signal transducer and activator of transcription
STING: stimulator of interferon genes
TPM: transcripts per kilobase million
TRF: telomere restriction fragment

## 1 INTRODUCTION

The stability of cellular DNA is challenged by DNA damaging agents, most of which are metabolic by‐products such as reactive oxygen species (ROS), and occasionally exogenous agents such as environmental chemicals and radiations. These agents induce tens of thousands of DNA lesions in a cell each day (Tubbs & Nussenzweig, 2017). The response to this ceaseless assault on genome integrity is the DNA damage response (DDR)—a broad, multi‐tiered signal transduction network that activates lesion‐specific DNA repair pathways while modulating numerous cellular circuits (Chatterjee & Walker, 2017). The rapid, highly structured, and fine‐tuned DDR is vigorously activated by DNA double‐strand breaks (DSBs) (Goldstein & Kastan, 2015). Genetic defects that ablate important DDR components lead to severe genome instability disorders (Taylor et al., 2019). Among their hallmarks are progressive degeneration of specific tissues, cancer predisposition, and segmental premature aging, highlighting the genome instability‐aging link. Importantly, combinations of sequence variations in DDR genes play a role in the broad range of morbidity in the general population, including differences in aging pace and aging‐associated diseases (Yousefzadeh et al., 2021).

The autosomal‐recessive genome instability disorder, ataxia‐telangiectasia (A‐T) is caused by null alleles in the ATM gene (Savitsky et al., 1995), which encodes the ATM protein kinase. A‐T is characterized primarily by progressive cerebellar degeneration, oculocutaneous telangiectasia, chronic lung disease, predisposition to malignancies, immunodeficiency, chromosomal instability, acute sensitivity to ionizing radiation (IR), and segmental premature aging (Rothblum‐Oviatt et al., 2016). The premature aging component of A‐T recently gained special attention, and probably contributes to many A‐T symptoms (Aguado et al., 2022; Shiloh & Lederman, 2017).

ATM is a homeostatic serine–threonine kinase whose most documented role is mobilizing the complex DSB response by phosphorylating numerous substrates in its many branches (Shibata & Jeggo, 2021). ATM is activated by DNA DSBs (Bakkenist & Kastan, 2003) as well as by ROS (Guo et al., 2010), in its capacity as a player in the cellular response to oxidative stress. It also plays a role in maintaining mitochondrial homeostasis and several other metabolic pathways (Lee & Paull, 2021).

Poor growth in culture of primary skin fibroblast lines from A‐T patients was reported early on (Elmore & Swift, 1976; Hoar, 1975). However, it was subsequently found that the initial growth of A‐T fibroblast lines at early passage levels was comparable to that of control cell lines, but A‐T cells senesced much earlier than controls (Shiloh et al., 1982)—an observation that was subsequently confirmed Davis & Kipling, 2009 and references therein. The premature senescence of primary A‐T fibroblasts was attributed to another hallmark of these cells—accelerated telomere shortening (Metcalfe et al., 1996).

Cellular senescence is a usually irreversible condition that includes cell cycle arrest, marked alterations in chromatin organization, genome stability, transcriptome dynamics, and numerous metabolic circuits, and a multi‐faceted senescence‐associated secretory phenotype (SASP) (Wiley & Campisi, 2021). Primary cell lines undergo replicative senescence after certain passage levels (Hayflick, 1998), but senescence can also be induced by oncogene activation, oxidative or genotoxic stresses, mitochondrial dysfunction, nutrient deprivation, interference with proteostasis, cell cycle inhibition, and epigenetic modifiers (Shmulevich & Krizhanovsky, 2021). The SASP is highly variable, dynamic and cell type‐dependent, leading to the secretion of a broad variety of bioactive molecules including pro‐inflammatory cytokines, and other factors with paracrine effects. The SASP can thus enable senescent cells to markedly affect their tissue environment. While cellular senescence plays important roles in developmental and tissue repair processes and eliminates damaged cells from tissues, thus serving as a barrier against neoplasia, evidence is mounting that accumulation of senescent cells in aging tissues is associated with tissue dysfunction, and aging‐associated morbidity (Di Micco et al., 2021).

Accelerated cellular senescence in certain tissues might contribute to the complex, progressive A‐T symptomatology. We asked whether the premature senescence of cultured A‐T fibroblasts is similar to the replicative senescence (RS) of fibroblasts from control donors, only accelerated, or it has other, unique characteristics. In view of ATM's role in maintaining the cellular redox balance, we asked whether bringing oxygen concentration closer to physiological levels (Keeley & Mann, 2019) might relieve this phenotype. If not, would accelerated senescence of A‐T cells under physiological oxygen levels still reflect the pathway dynamics characterizing senescing control cells? We addressed these questions by monitoring the growth and senescence parameters of A‐T and control fibroblast lines at ambient versus 3% oxygen concentration, followed by comprehensive transcriptomic and pathway analyses.

## 2 RESULTS

### 2.1 The accelerated senescence of primary A‐T fibroblasts is retained at physiological oxygen concentration

**FIGURE 1:**
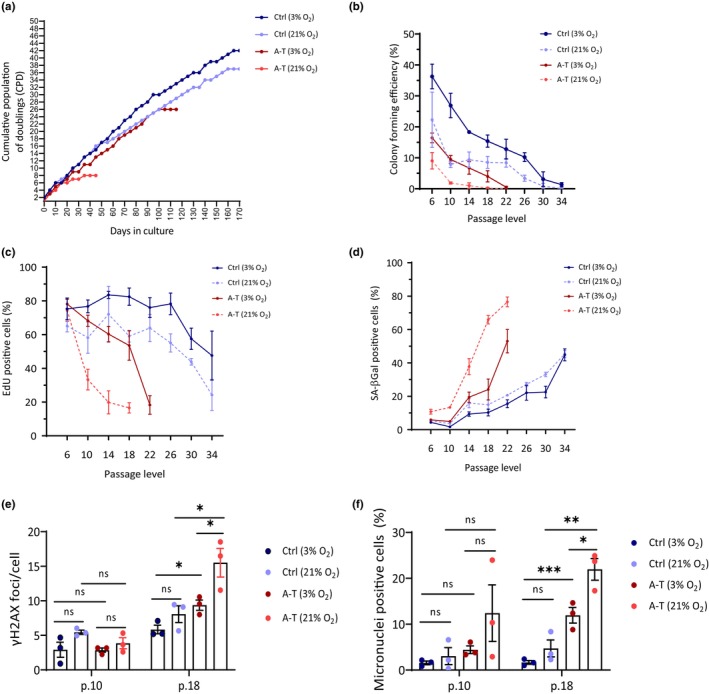
Characterization of cellular senescence in primary skin fibroblasts from A‐T patients and controls under physiological versus ambient oxygen concentrations. (a) Cumulative population doubling (CPD) curves of a control (Ctrl; a healthy donor) and an A‐T fibroblast line as a function of time in 21% and 3% O_2_ concentrations. (b) Quantification of colony‐forming efficiency of three control and three A‐T fibroblast lines as a function of passage level, in 21% and 3% O_2_. (c) Quantification of EdU‐positive cells in the same experiment as in B. (d) Quantification of SA‐β‐Gal‐positive cells in the same experiment. (e) Amounts of γH2AX nuclear foci in the same experiment. (f) Amounts of micronuclei‐positive cells in the same experiment. (*t*‐test, *n* = 3).

Six control and six A‐T primary skin fibroblast lines were used in this study (Table S1). All donors were unrelated to each other. Western blotting analysis (not shown) indicated that all A‐T cell lines were devoid of ATM protein, like in most A‐T patients (Gilad et al., 1996), due to either homozygosity or compound heterozygosity for null *ATM* alleles (Table S1). We monitored growth rate and senescence readouts in three control and three A‐T cell lines (Table S1), which were cultured at either ambient or 3% oxygen concentrations. Compared to ambient oxygen, 3% O_2_ improved the ability of both A‐T and control cell lines to thrive and extended their lifespan in culture, with markedly higher improvement in A‐T cells (Figures 1a and S1). Despite this general improvement, at 3% O_2_ A‐T cell lines still stopped proliferating earlier than control cell lines in terms of cumulative population doublings (CPD) or passage levels (Figures 1a and S1). At both oxygen levels, other proliferative readouts such as colony forming efficiency (CFE) and rate of DNA synthesis declined earlier in A‐T cells compared to controls (Figure 1b,c). Furthermore, the fraction of senescence‐associated β‐galactosidase (SA‐β‐Gal)‐positive cells—a major senescence hallmark—increased earlier in A‐T cells (Figure 1d). Two readouts of DNA damage and genome instability—amounts of γH2AX nuclear foci and appearance of cytoplasmic micronuclei—were reduced in both genotypes at 3% O_2_ compared to ambient O_2_ but remained higher in A‐T cells compared to controls (Figure 1e,f and S2). Collectively, these results suggested that, despite the enhancement of proliferation and the marked delay of cellular senescence conferred by growth in 3% oxygen, an inherently premature senescence remained a prominent characteristic of primary A‐T skin fibroblasts.

Primary A‐T fibroblasts exhibit accelerated telomere shortening (Metcalfe et al., 1996; Pandita, 2001). Notably, this characteristic was retained under 3% oxygen (Figure S3). Since under this oxygen concentration A‐T cells could reach considerably higher passage levels compared to under ambient atmosphere (Figure 1a), by the time they finally senesced their telomeres were markedly eroded (Figure S3). Notably, the rapid premature senescence of A‐T fibroblasts under ambient oxygen, with telomeres considerably longer than those of cells growing under 3% oxygen, suggests that telomere shortening was not the primary cause of this premature senescence but rather alterations in various physiological circuits.

### 2.2 Transcriptome dynamics in A‐T fibroblasts senescing at physiological oxygen levels: Unique and common patterns

#### 2.2.1 Generation of an RNA‐seq dataset

**FIGURE 2:**
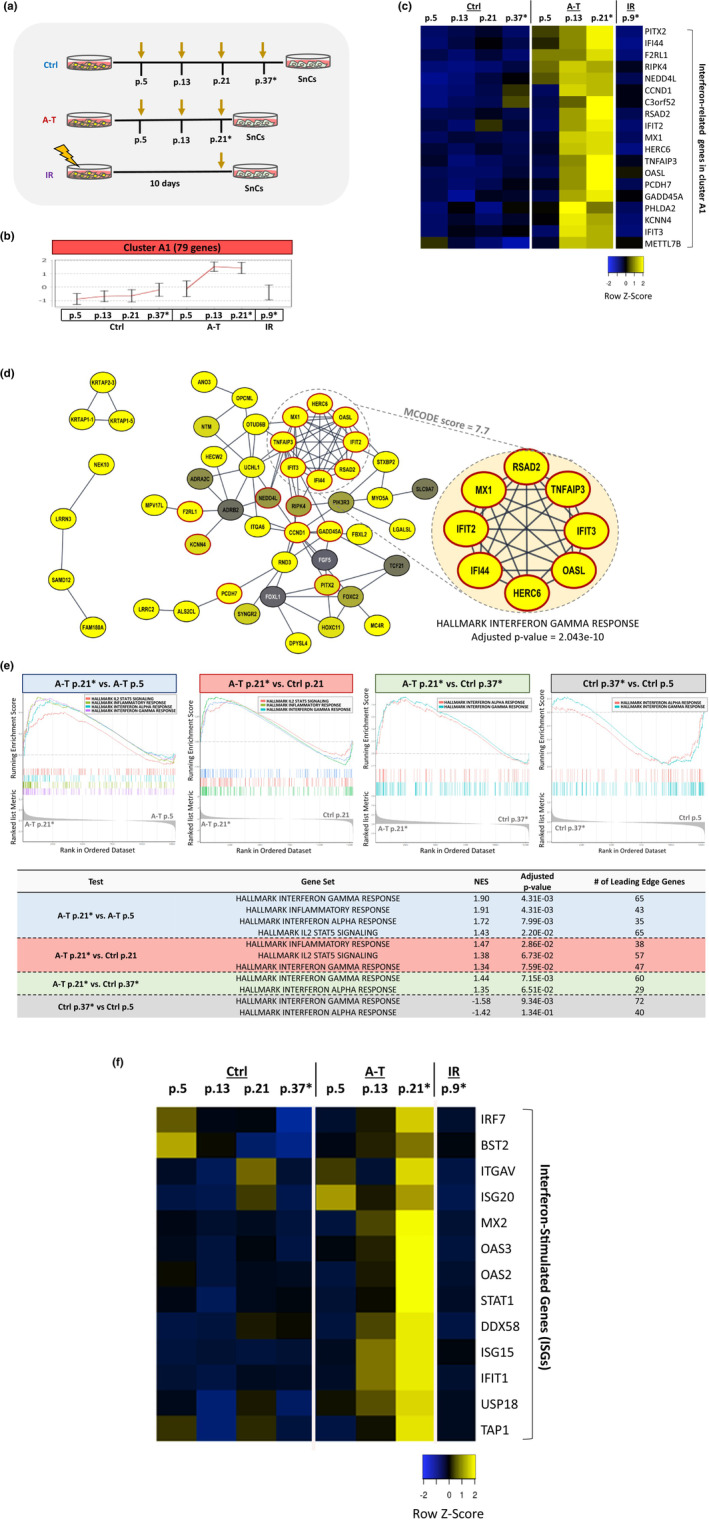
Up‐regulation of the interferon pathway in primary A‐T fibroblasts senescing in 3% oxygen. (a) Schematic experimental workflow. (b) Gene expression cluster A1 (Figure S3) representing genes whose expression sharply rises in A‐T cells around p. 13. The X‐axis represents the different conditions defined by genotypes and passage levels. The Y‐axis shows normalized gene expression levels. The red trend‐line passes through the mean expression values corresponding to each condition, with STD error bars shown. Asterisk (*) indicates senescing cells. (c) Heat map of 19 selected, functionally significant genes included in Cluster A1. The figure shows the normalized (transcripts per kilobase million, TPM) expression level of each gene. Blue represents down‐regulation, yellow represents up‐regulation. The chosen genes are all identified as part of the interferon pathway or interferon targets. Asterisk (*) indicates senescing cells. (d) STRING protein–protein interaction (PPI) network of proteins encoded by genes in cluster A1. The node colors denote the log_2_ of expression fold change of the late passage level (p. 21) versus the early passage level (p. 5) in A‐T cells. Red‐circled nodes correspond to the genes present in the interferon‐related heat map in C. The highlighted subnetwork corresponds to the densest region of the network according to the MCODE algorithm and is entirely part of the interferon pathway. (e) Global gene set enrichment analysis (GSEA) for Interferon and Interferon‐related gene sets. Top panel are the GSEA plots. Below is an enrichment table, in which the enriched Hallmark gene set and normalized enrichment score (NES) are shown in columns 2 and 3, respectively. Adjusted *p*‐value and number of leading‐edge genes are in columns 4 and 5. Asterisk (*) indicates senescing cells. (f) A heat map of selected ISGs presenting their relative expression in A‐T, control and IR‐treated cells.

RNA‐seq analysis was carried out on three control and four A‐T cell lines (Table S1). The cells were grown in 3% O_2_ and sampled at different passage levels that represent their genotype‐specific senescence progression (Figure 2a). In view of the presumed role of DNA damage in the accelerated senescence of A‐T cells, we added to this analysis a commonly used standard of DNA damage‐induced senescence: three control fibroblast lines that had been irradiated with 10 Gy of X‐ray and subsequently allowed to senesce for 10 days (Figure 2a). The analysis encompassed a total of 19,792 genes whose expression was readily detected, of which 12,813 were protein‐coding. Principal component analysis (PCA) of the entire gene set (*n* = 19,792) revealed global transcriptomic trends based on shared gene expression patterns (Figure S4A). It confirmed that the experimental conditions defined by genotype, passage level, and radiation treatment dominantly affected gene expression patterns. Similarly, hierarchical clustering showed a marked separation of transcriptional patterns between genotypes, but the separation based on passage levels was less definite in the entire gene set (Figure S4B). In order to retrieve DEGs in various conditions, we applied a multiple comparison test. A total of 2625 DEGs were identified (Table S2). Each comparison test was followed by a separation criterion to filter out genes with unstable expression across replicates (see Methods). Finally, a total of 2299 DEGs were found to be significantly up‐ or down‐regulated across the various experimental conditions (Table S2). Using real‐time PCR (qPCR) we validated the transcriptional dynamics reflected in the high‐throughput analysis of several DEGs representing different functional groups (see below) (Figure S5). Further analysis was based on this DEG set unless noted otherwise.

#### 2.2.2 Identification of gene expression patterns

Genes with similar expression patterns are often functionally related. We therefore clustered the genes in the final DEG set according to shared temporal expression patterns across passage levels and genotypes. Twenty‐eight clusters with distinct expression patterns were obtained and were divided into six groups according to their similarities (Figure S6).

#### 2.2.3 Physiological pathways represented in gene clusters with A‐T‐specific expression patterns

Gene ontology (GO) enrichment analysis was used to reveal the corresponding physiological circuits in specific clusters. In cluster A1 (genes with expression levels sharply rising in A‐T cells around passage level 13, Figures 2b and S6), a highly significant functional group represented the interferon (IFN) response, with classical IFN‐stimulated genes (ISGs) (e.g., *MX1*, *IFIT2*, *IFIT3*, *IFI44*, and *TNFAIP3*) strongly induced (Figures 2c and S7A; Table S3). Imposing these genes onto a protein–protein interaction (PPI) network demonstrated extensive interactions among their protein products (Figure 2d). The results were further corroborated by a global gene set enrichment analysis (GSEA) using all protein‐coding genes in the dataset (Figure 2e). Applying GSEA to low versus high passage A‐T cells as well as high‐passage A‐T versus high‐passage control cells showed enrichment of the IFN response and high expression of several ISGs (*MX2*, *OAS2*, *OAS3*, *IFIT1*, *DDX58*, *TAP1*, *USP18*, *ITGAV*, *BST2*, *ISG15*, and *ISG20*) as well as genes encoding the pattern recognition receptor (PRR) effectors (STAT1 and IRF7) that regulate the expression of ISGs (Figures 2e,f and S7A). These observations were confirmed using a public dataset (GEO accession GSE35347) (Nayler et al., 2012). In that study, the authors used human primary skin fibroblasts from controls and A‐T patients growing in ambient oxygen concentration, to study the effect of their *ATM* genotype on their conversion to pluripotent stem cells. We performed a GSEA analysis on that dataset (Figure S8). Gene expression data were clustered using a UMAP analysis, illustrating the quality and relevance of these data (Figure S8A). The interferon response was found as a top hit in this GSEA analysis (Figure S8B), confirming the link between ATM depletion and the interferon response pathway. Indeed, elevated expression of the majority of these ISGs in senescing cells was previously documented ([Mullani et al., 2021] and references therein). Interestingly, only a few of them were moderately increasing in senescing control cells (Figures 2f and S7A). Therefore, these findings highlight a feature of senescing cells that is profoundly expressed in A‐T cells senescing under physiological O_2_ concentration. Strikingly, IFNs themselves were not expressed in A‐T fibroblasts. The robust activation of IFN response along with the ISGs activation in the absence of IFN expression was also observed in a previous study on the premature aging disease, Hutchinson‐Gilford progeria syndrome (HGPS) (Kreienkamp et al., 2018), suggesting that the upregulation of ISGs in senescing A‐T fibroblasts, similar to HGPS fibroblasts, is probably a cell‐intrinsic and IFN‐independent process.

**FIGURE 3:**
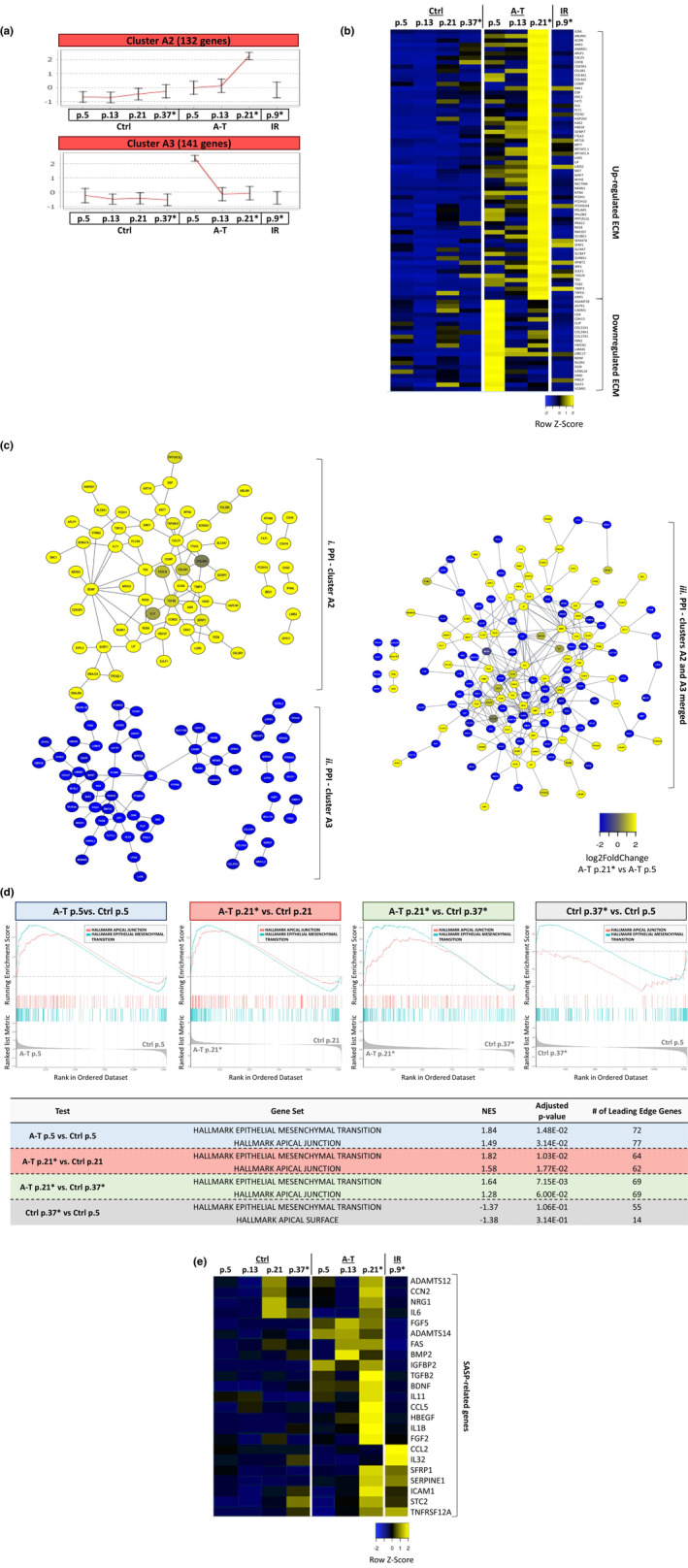
Altered ECM regulation and up‐regulated SASP in senescing A‐T fibroblasts. (a) Gene expression clusters A2 and A3. (b) A heat map of functionally significant genes included in clusters A2 and A3 (83 genes total, 62 from cluster A2 and 21 from A3). The figure represents the normalized (TPM) expression level of each gene. These genes were identified as part of the ECM. (c) STRING PPI networks corresponding to clusters A2 and A3. The two upper networks one includes genes only from i. cluster A2 and ii. cluster A3. The above iii. network is a superposition of both. The different colors of the nodes are based on log_2_ of the fold‐change in gene expression at a late passage level (p. 21) versus early passage level (p. 5) in A‐T cells. (d) GSEA plots and table for ECM and ECM‐related gene sets. NES: normalized enrichment scores. (e) Representative heat map of SASP‐related gene expression comparing A‐T, control and IR‐treated cells.

Other notable clusters were A2, which included genes whose expression rose in senescing A‐T cells (Figures 3a,b and S6), and A3, A4, and C3 with genes whose expression declined in senescing A‐T cells (Figures 3a,b and S6). Surprisingly, both the up‐ and down‐regulated genes in these clusters take part in similar processes, mostly related to extracellular matrix (ECM) remodeling and organization (Figure 3b and Table S3). Furthermore, the PPI map that corresponds to a combined list of the up‐ and down‐regulated ECM genes shows that the protein products of these genes all fall into the same interaction map (Figure 3c), suggesting that they are functioning and interacting together. The ECM genes include those encoding for insoluble and structural components (collagens and laminins) as well as soluble and secreted factors (TIMP3, TGFb2, IGFBP7, LIF, IL11, and TGFb2) and shed receptors (PTGER3, ITGB1/5, and ITGA3/6) (Figure 3b). The ECM alterations in A‐T cells were also reflected on a global scale using GSEA (Figures 3d and S7B). Interestingly, all the GSEA comparisons pointed to enrichment of the epithelial‐to‐mesenchymal transition (EMT) in senescing A‐T cells (Figure 3d), which was previously observed in A‐T cells in ambient oxygen (GEO GSE35347 dataset; Figure S8C). EMT is a process that reflects massive ECM deposition that is known to be enhanced by the SASP and is recognized as a key driver of age‐related fibrosis and tumorigenesis (De Blander et al., 2021). Indeed, many of the up‐regulated ECM genes are also part of the SASP (*LIF*, *DKK1*, *SPP1*, *TGFB2*, *IL11*, and *IGFBP7*; Figure 3b). Increased expression of these SASP genes in senescing cells has been documented, and prematurely senescing A‐T cells seem to be no exception. Elevated expression of additional SASP genes in A‐T cells was also observed when we looked at the entire dataset (e.g., *ADAMTS12/14*, *CCN2*, *SERPINE1*, *TNFRSF12A*, *IGFBP2*, *BDNF*, *SFRP1*, *STC2*, *BMP2*, *IL32*, *IL1B*, *IL6*, *FGF2/5*, *NRG1*, *CCL2*, *CCL5*, *HBEGF*, *FAS*, *and ICAM1*) (Figures 3e and S7B). Expression of some of the SASP factor genes was moderately or highly rising also in control cells at advanced passage levels, or after irradiation, but some of them were specific for A‐T cells and may be part of their unique SASP profile (Figures 3e and S7B).

**FIGURE 4:**
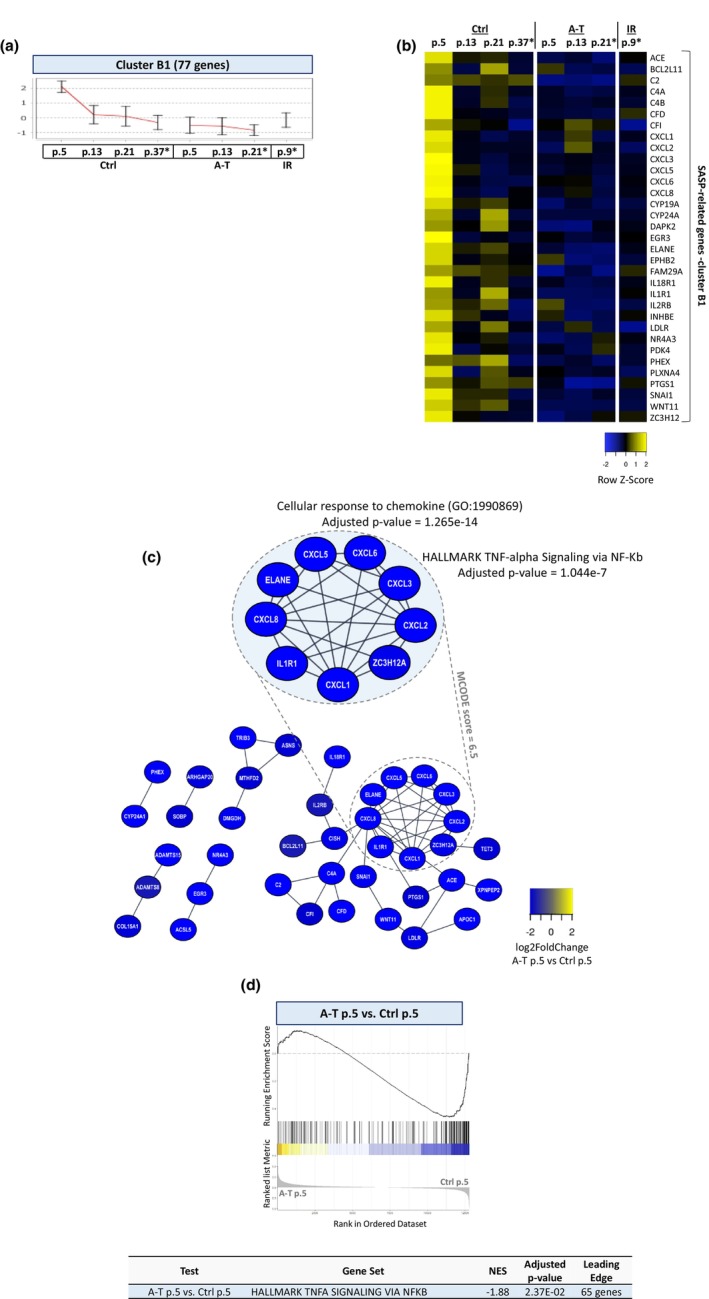
Part of the SASP is down‐regulated in senescent A‐T fibroblasts under physiological oxygen concentrations. (a) Gene expression cluster B1. (b) A heat map of 33 SASP‐related genes included in cluster B1. (c) STRING PPI network of the proteins encoded by the genes in cluster B1. The different colors of the nodes are based on log2 of the fold‐change in gene expression of A‐T cells at early passage (p. 5) versus control cells at early passage (p.5). The highlighted sub‐network corresponds to the densest region of the network according to the MCODE algorithm, which represents parts of the TNF‐alpha signaling via NF‐κB and the cellular response to chemokines. (d) Complementary GSEA for the SASP‐related NF‐κB gene set.

On the other hand, other pro‐inflammatory SASP genes were constitutively repressed in A‐T cells (Cluster C3, Figure 4a). That is, while their expression level was down‐regulated as the control cells progressed through passages and in response to IR, it was completely repressed in A‐T cells already at the early passages. Interestingly, the majority of them belong to the CXCL chemokine family (e.g., *CXCL1*, *CXCL2*, *CXCL3*, *CXCL5*, *CXCL6*, *and CXCL8*) (Figure 4b), and their encoded proteins were highly interacting (Figure 4c). GSEA analysis on A‐T versus control cells at an early passage level showed down‐regulation of the NF‐κB signaling pathway (Figure 3d). Importantly, these SASP factors were gradually declining in control cells as they advanced in passage levels and were repressed upon radiation‐induced senescence (Figure 4a). Overall, the results demonstrated a unique SASP in prematurely senescing A‐T fibroblasts growing in physiological oxygen concentrations, along with deregulation of genes encoding ECM components.

**FIGURE 5:**
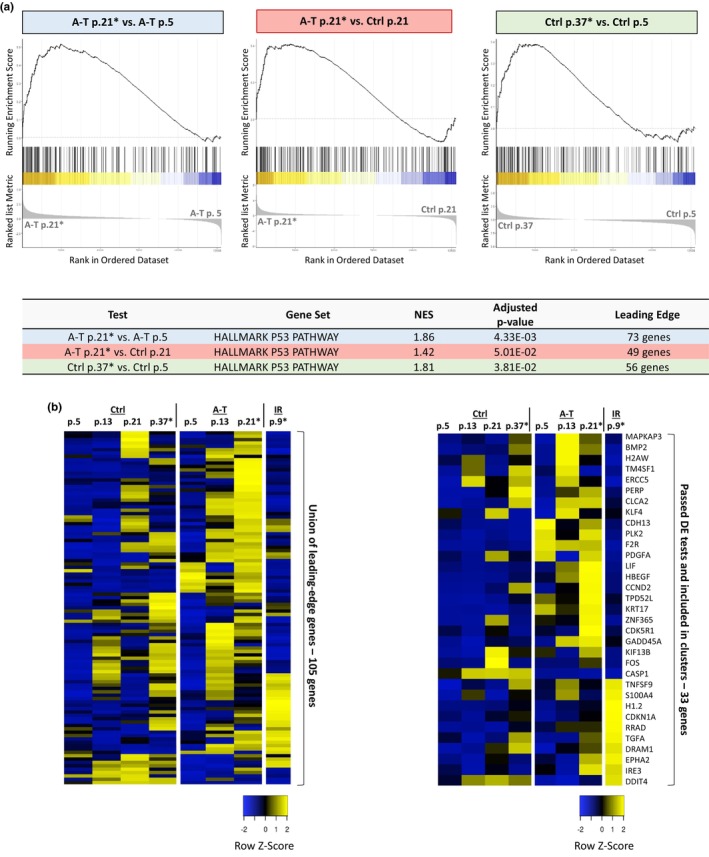
Global GSEA identifies enrichment patterns of p53 target genes. (a) GSEA plots and table related to p53 target genes. Asterisks (*) denote senescing cells. (b) Heat maps showing expression levels of selected genes in p53‐mediated signaling. The heat map on the left represents a union of 105 leading‐edge genes and the right map includes 33 of the leading‐edge genes that passed DE and separation tests. The figure is based on normalized Transcripts per Kilobase Million (TPM) gene expression levels. Asterisks (*) denote senescing cells.

Using GSEA, we examined the behavior of genes in the p53‐mediated response pathway in our dataset. Several p53‐target genes were up‐regulated in a passage‐dependent manner in both A‐T and control cells (Figure 5a), indicating involvement of the p53 pathway in senescence progress in both genotypes. Similar results were obtained in A‐T cells in the GEO GSE35347 dataset (Figure S8D)—a notable finding in view of the absence in A‐T cells of the ATM protein, p53's major upstream regulator in the response to DSBs. A closer examination of these genes revealed many p53 target genes including the p21^Wai1/CiP1^ protein, a pleiotropic inhibitor of cyclin/cyclin‐dependent kinase complexes that mediate cell cycle progression (Figure 5b). These results suggest that the p53‐p21 axis might be activated in senescing A‐T cells growing at low oxygen levels, but with a unique gene expression signature.

Another senescence axis is the 16INK4a–pRb pathway (Di Micco et al., 2021). Increasing expression of the *CDKN2A* and *CDKN2B* genes encoding the p16 and p15 proteins, respectively, was observed in senescing control and A‐T cells but less so in irradiated cells. Similarly, *RB1* expression rose during senescence in both genotypes but remained relatively low following irradiation. These results suggest involvement of the 16INK4a–pRb axis in mediating the cell cycle arrest in both A‐T and control cells undergoing senescence, and points to the similarity of replicative senescence in control and A‐T cells, which is distinctive from that of IR‐treated cells, which relies heavily on the activation of the p53–p21 axis.

#### 2.2.4 Overall comparison of transcriptomic patterns

**FIGURE 6:**
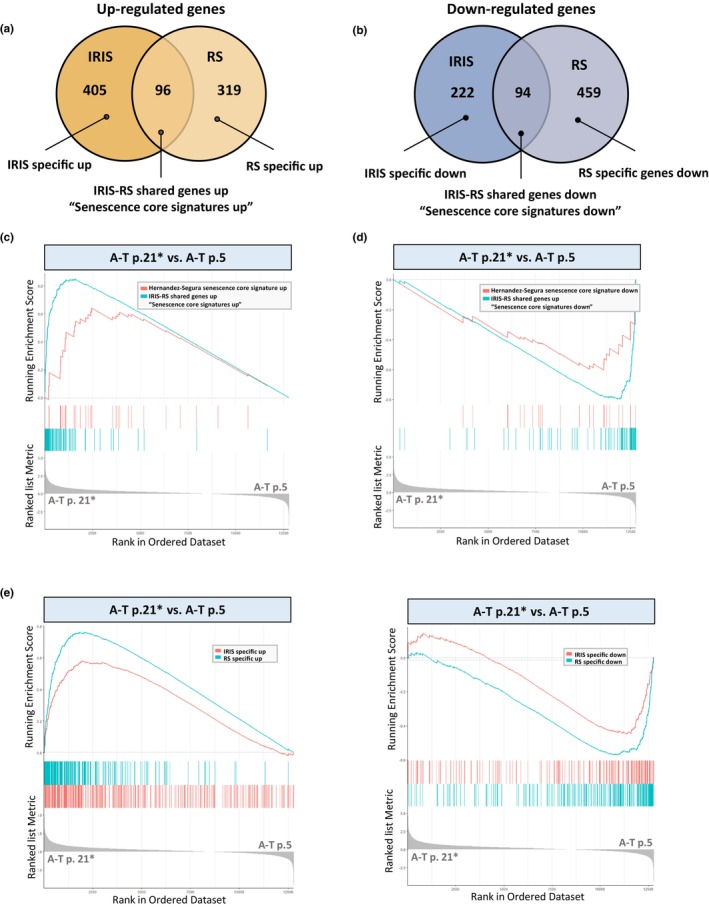
Senescent A‐T cells exhibit replicative senescence‐like gene expression signature. (a) Venn diagram showing the overlap of differentially up‐regulated genes in control cells undergoing RS or IRIS. (b) Venn diagram showing the overlap of differentially down‐regulated genes in the same cells as in A. (c) GSEA plots for senescent A‐T cells (p. 21) compared to proliferative A‐T cells (p. 5). The blue curve represents the “RS‐IR shared genes up” signature from the overlap region in (a) and the red curve represents the Hernandez‐Segura et al. (2017) up‐regulated senescence signature; both are positively enriched (up‐regulated) in senescing A‐T cells. (d) GSEA plots comparing senescent A‐T cells (p. 21) compared to proliferative A‐T cells (p. 5). The “RS‐IR shared genes down” signature from the overlap region in (b) (blue line) and the Hernandez‐Segura down‐regulated senescence signature (red line) are both negatively enriched (down‐regulated) in senescing A‐T cells. (e) GSEA plots for senescent A‐T cells (p. 21) compared to proliferative A‐T cells (p. 5). The type‐specific up‐regulated signatures in senescence (“IR‐up” or “RS‐up”) were derived from the non‐overlapping parts of the Venn diagram in (a). The “RS‐up” signature (blue line) shows a greater positive correlation (NES = 2.79, Adjusted *p*‐value = 1.33E‐10) than the “IR‐up” signature (red line) (NES = 2.1, Adjusted *p*‐value = 1.33E‐10). (f) GSEA plots for senescent A‐T cells (p. 21) compared to proliferative A‐T cells (p. 5). The type‐specific down‐regulated signatures in senescence (“IR‐down” or “RS‐down”) were derived from the non‐overlapping parts in the Venn diagram in (b). The “RS‐down” signature (blue line) shows a greater negative correlation (NES = −2.81, Adjusted *p*‐value = 1.33E‐10) than the “IR‐down” signature (red line) (NES = −2.22, Adjusted *p*‐value = 3.99E‐10).

The irradiated control cells allowed us to compare the transcriptomic dynamics associated with RS at 3% oxygen and IR‐induced senescence (IRIS) of these cells. When we compared the transcriptomes of ‘young’ (passage 5) versus senescing (passage 35) control cells, we identified 968 DEGs (415 up‐regulated and 553 down‐regulated). Similar comparison of the vigorously growing, early passage control cells and the same cells senescent after irradiation identified 817 DEGs (501 up‐regulated and 316 repressed). The DEG lists shared 96 up‐regulated (Figure 6a) and 94 down‐regulated genes (Figure 6b), which we regarded as a core of senescence‐associated transcriptomic signatures. These core signatures were used in global GSEA comparing actively growing ‘young’ A‐T cells (passage 5) and senescing A‐T cells (passage 21). Importantly, both parts of this core (up‐ and down‐regulated genes) were significantly enriched in this GSEA (Figure 6c,d), and the enrichment directions (up‐ or down‐regulation) were similar in control and A‐T cells. These results further demonstrate that the transcriptome dynamics in prematurely senescing A‐T cells is similar in many respects to that of RS or IRIS in control cells. Notably, both parts of this core (up‐ and down‐regulated genes) were significantly enriched when we compared A‐T cells at passage 21 with control cells at the same passage (data not shown), highlighting the premature aspect of the senescence of cells devoid of ATM.

We wondered how the current A‐T data obtained at 3% O_2_ would compare with senescence signatures obtained in senescing human cells grown at ambient oxygen. For this purpose, we carried out a parallel GSEA using a previous comprehensive dataset obtained in human fibroblasts, HCA2 cells, keratinocytes, and melanocytes growing in ambient oxygen (Hernandez‐Segura et al., 2017). The senescence‐associated core signature in that dataset was subdivided into up‐ and down‐regulated gene sets. Here too, GSEA showed the significant enrichment of the Hernandez‐Segura et al. (2017) core signature in our dataset, in similar directions (up‐ or down‐regulated) (Figure 6c,d). This result suggests that the senescence of our cells at 3% oxygen shares common features with that observed at ambient oxygen.

We then turned to genes whose expression patterns are not included in the shared portions of the Venn diagrams in Figure 6a,b. Their expression patterns were therefore specific to either RS or IRIS. The RS‐ and IRIS‐specific gene sets were used in global GSEA comparing passages 5 and 21 of A‐T cells. The results indicated that both up‐ and down‐regulated genes generally behaved similarly in senescing A‐T cells (Figure 6e). Interestingly, genes up‐regulated in RS showed higher enrichment in A‐T cells (NES = 3.37) than IRIS up‐regulated genes (NES = 2.28). Similarly, down‐regulated genes in RS were also down‐regulated in senescing A‐T cells at higher significance (NES = −2.26) than down‐regulated IRIS genes (NES = −2.07). This analysis therefore revealed closer transcriptomic proximity between senescing A‐T cells and control cells in RS compared to irradiated control cells.

Collectively, the results suggest that the transcriptomic dynamics in A‐T cells senescing prematurely at 3% O_2_ shares patterns with RS and IRIS of control cells at both physiological and ambient oxygen levels, with higher similarity to RS.

## 3 DISCUSSION

Understanding many A‐T symptoms remains a challenge, particularly in view of ATM's expanding roles in cellular metabolism (Lee & Paull, 2021). The contribution of premature aging to A‐T symptomatology presumably increases as the patients advance in age (Aguado et al., 2022; Shiloh & Lederman, 2017). The premature senescence of A‐T fibroblast lines probably represents this component of the disease in the culture dish. A major difference between the in vivo tissue environment and the incubator setting is the oxygen concentration (Keeley & Mann, 2019). The extended lifespan of primary cell lines obtained by lowering the surrounding oxygen concentration is an established observation (Chen et al., 1995; Parrinello et al., 2003). The combination of elevated endogenous DNA damage and impaired redox balance, both emanating from ATM's absence, is probably an important driver of the accelerated senescence of primary A‐T fibroblasts growing in ambient oxygen concentration. Indeed, the improved lifespan and proliferation rate under reduced oxygen level was relatively higher in A‐T cells than in control cells, but a marked difference still remained between the two genotypes with regard to lifespan until senescence, suggesting additional causes of this fundamental phenotype of A‐T fibroblast lines. We therefore undertook to identify cellular pathways at the core of this A‐T phenotype without its exacerbation by ambient oxygen levels. We asked whether the premature senescence of primary A‐T fibroblasts at 3% O_2_ exhibits similar characteristics to those of replicative senescence of control cells, which occurs at later stages of growth in culture, or whether A‐T cells might show different senescence‐associated reflected in transcriptomic patterns.

The predominant group of ISGs, whose expression rose in senescing A‐T cells, does indeed reflect a well‐documented component of replicative senescence (Frisch & MacFawn, 2020). Notably, the rise in ISG expression was more moderate in control cells, emphasizing their vigorous expression in senescing A‐T cells. A crucial inducer of the interferon response is the cGAS‐STING signaling axis, whose major canonical activator is cytoplasmic DNA, which can be generated by excessive DNA damage (Ritchie et al., 2022). A primary mechanism leading to the presence of genomic DNA fragments in the cytosol is micronuclei (MN) formation during mitosis. MN eventually rupture spilling their DNA content into the cytosol thereby activating the cGAS‐STING cytosolic DNA‐sensing pathway (Bakhoum et al., 2018; Dou et al., 2017; Harding et al., 2017; MacDonald et al., 2023). Indeed, we observed elevated numbers of micronuclei in our senescing A‐T fibroblast lines, under both oxygen concentrations. Accumulation of cytoplasmic DNA and subsequent activation of cGAS‐STING, along with interferon production and ISGs activation, were previously observed in A‐T cells growing under ambient oxygen Aguado et al., 2021 and references within. Moreover, pharmacological inhibition of either cGAS or STING in A‐T brain organoids ameliorated many senescence signatures and improved neuronal synaptic activity and survival in these organoids (Aguado et al., 2021).

Importantly, in our experiments, the expression of interferon genes themselves was not elevated in senescing control or A‐T fibroblasts despite the elevation of ISGs expression. Notably, a previous study showed that hypoxia led to transcriptional and translational down‐regulation of the interferon genes (Miar et al., 2020). The canonical pathway downstream of interferons finally culminating in ISGs activation involves the stimulation of the interferon receptors, IFNAR1/IFNAR2, and subsequent activation of the STAT1/2 proteins (Borden et al., 2007). Importantly, our data do show elevated STAT1/interferon‐like response in addition to ISG expression in senescing A‐T cells. Similarly, primary fibroblasts from Hutchinson‐Gilford progeria (HGP) patients were found to exhibit a robust interferon‐like response, but the interferons themselves were not expressed in these cells. Furthermore, the cGAS‐STING pathway could activate STAT1 and upregulate the ISG expression in HGP cells via a cell‐intrinsic non‐canonical pathway that was independent of interferons expression (Kreienkamp et al., 2018). Collectively, our findings suggest that in A‐T cells growing under 3% oxygen, an intrinsic non‐canonical activation of STAT1/interferon‐like response may act downstream of cGAS‐STING, ending in ISG induction. Given the involvement of the cGAS‐STING pathway in inflammation, senescence and disease, inhibitors of cGAS (Lama et al., 2019) or STING (Haag et al., 2018) could potentially be used to treat age‐related chronic inflammation in A‐T patients.

Another major functional group of genes that was modulated in a distinct manner in senescing A‐T cells was the ECM. Aberrant ECM deposition has been observed in senescent cells, and contributes to various age‐related pathologies, including chronic fibrotic diseases, and cardiovascular diseases (Blokland et al., 2020; Levi et al., 2020). Importantly, while certain ECM genes were sharply down‐regulated in senescing A‐T fibroblasts, many others exhibited enhanced expression as A‐T cells were advancing towards senescence. Moreover, the protein products of both up‐regulated and down‐regulated genes in this group function in the same processes, suggesting severe disarray in ECM dynamics in these cells. These proteins take part in the formation of insoluble structural factors (e.g., collagens) as well as soluble and secreted factors. Importantly, several soluble ECM components are also SASP factors (Birch & Gil, 2020; Roger et al., 2021), and indeed among the up‐regulated genes were those encoding the SASP factors, DKK1, SPP1, CCN2, the pro‐fibrotic TGFβ2 factor, and the TGFβ downstream effectors, SERPINE1, IL11, and IGFBP7 (Han et al., 2022). In addition to promoting senescence in a paracrine manner, TGFβ2, IL11, and SERPINE1 can promote epithelial to mesenchymal transition (EMT) and fibrosis [reviewed in (Lovisa, 2021)].

While the expression of several SASP genes rose in senescing A‐T cells, that of other pro‐inflammatory SASP genes remained stably low in senescing control and A‐T cells as well as the irradiated cells. A prominent class in this group was the CXCL chemokine family, which is often over‐expressed in senescent cells (Coppe et al., 2010; Lopes‐Paciencia et al., 2019). Notably, the SASP composition is highly heterogeneous among cell types and reflects epigenetic and environmental variations (Hernandez‐Segura et al., 2018). The SASP pattern that we observed might characterize fibroblasts grown at physiological oxygen concentrations. Interestingly, two recent studies in murine cultured cells and tissues showed that low oxygen levels or treatment with hypoxia‐mimetic compounds reduces the activity of the transcription factor NF‐κB and the secretion of several downstream pro‐inflammatory SASP factors (Seno et al., 2018; van Vliet et al., 2021), and expression of the CXCL family is indeed regulated by NF‐κB (Coppe et al., 2010; Lopes‐Paciencia et al., 2019).

Genes encoding SASP factors, such as DKK1, SPP1, and IGFBP7, were up‐regulated in our senescing A‐T fibroblasts. These genes are driven by p53—another apical regulator of cellular senescence (Lopes‐Paciencia et al., 2019) via its downstream effector, p21, which can provoke a specific type of SASP independently of NF‐κB (Sturmlechner et al., 2021). Of note, p53 is considered a negative regulator of NF‐kB‐dependent pro‐inflammatory SASP genes (Lopes‐Paciencia et al., 2019). Thus, under physiological oxygen levels, the NF‐κB‐mediated secretome might be inhibited in senescing A‐T cells while p53‐dependent pathways are up‐regulated. Collectively, these findings highlight the importance of the surrounding oxygen concentration as determinant for pro‐inflammatory SASP expression and composition.

In addition to its involvement in the SASP, p53 is a key regulator of the cell cycle checkpoints that are induced by DNA damage (most notably after DSB induction), following its ATM‐dependent activation and stabilization. p53 acts in this cardinal DDR pathway in its capacity as transcription regulator (Vaddavalli & Schumacher, 2022). Prolonged cell cycle arrest may lead to senescence (Sheekey & Narita, 2021). A central p53 target gene in this pathway is *CDKN1A*, which encodes the CDK inhibitor, p21^WAF1/CIP1^. Accordingly, p21 levels were markedly increased in our irradiated control cells, and this pathway was also up‐regulated in unirradiated, senescing A‐T cells, suggesting that accumulation of DNA damage plays a role in their premature senescence.

Another pathway with a central role in cellular senescence is the 16^INK4a^–pRb signaling axis (Di Micco et al., 2021). Interestingly, expression of the genes encoding p16 and p15 (*CDKN2A* and *CDKN2B*, respectively) as well as the *RB1* gene, was elevated in both senescing A‐T and control cells but not in the irradiated cells. This result suggests that the premature senescence of A‐T fibroblasts shares driving pathways with RS of control cells, which are not activated during the rapid senescence induced by acute DNA damage.

In sum, the transcriptome dynamics of A‐T cells showed greater similarity to that of replicative senescence of control cells than to that of irradiated cells. Furthermore, this dynamics showed strong similarity to the transcriptome of control human fibroblasts senescing in ambient oxygen concentration. This means that in physiological oxygen concentration, prematurely senescing A‐T fibroblasts mobilize gene expression dynamics that is basically similar to that of the common replicative senescence observed under ambient oxygen. We conclude that the accelerated senescence of A‐T skin fibroblasts is a major feature of their cellular phenotype, which is partly alleviated by physiological oxygen concentration but still persists as an inherent characteristic of these cells. It exhibits several molecular characteristics of RS but also unique transcriptomic patterns, most notably the complex ECM‐associated gene expression pattern, the interferon‐like response, and a unique SASP gene expression. We find that in the absence of ATM, the p53‐p21, and p16‐RB1 pathways are preserved but lead to a unique gene expression signature. Notably, several SASP factors whose genes are up‐regulated in senescing A‐T cells can induce senescence in a paracrine manner and promote EMT–a major process during fibrosis and tumorigenesis. Thus, accumulation of senescent cells in body tissues of A‐T patients probably underlies at least part of the premature aging observed in these individuals as they advance in age.

## 4 MATERIALS AND METHODS

### 4.1 Human primary fibroblast cultures and procedures

Human skin fibroblasts from A‐T patients and unrelated donors unaffected with A‐T were grown in complete Dulbecco's modified Eagle's medium (DMEM) at 37°C in 5% CO_2_ and ambient (21%) or 3% O_2_. Growth curves were constructed by seeding in triplicates 3.5 × 10^4^ cells/well in 12‐well plates and counting them after trypsinization every 24 h for 5 consecutive days. The number of population doublings (PDs) per passage was obtained by counting the cells after trypsinization according to the formula, PD = (*N*_f_/*N*_0_)log_2_, where *N*_0_ is the number of the initially seeded cells and *N*_f_ is the number of the cells at the following trypsinization. The number of CPD was calculated as the sum of PDs over passages. Colony formation efficiency was measured according to (Shiloh et al., 1982), and immunoblotting—according to (Jachimowicz et al., 2019). SA‐β‐gal activity was monitored using the Senescence Detection Kit (Biovision). SA‐β‐Gal–positive cells were scored in multiple fields. At least 100 cells were identified per condition.

EdU incorporation to cellular DNA was monitored using a Cell Proliferation Assay Kit (Millipore). Edu‐positive cells in randomly selected fields were counted using the ImageJ software (NIH). At least 100 cells were scored for each condition.

### 4.2 Telomere length analysis

In‐gel hybridization analysis was done as previously described (Lamm et al., 2009). Briefly, genomic DNA was digested with the HinfI restriction endonuclease, electrophoresed, denatured, and analyzed using in‐gel hybridization with a telomeric probe, (AACCCT)3, to visualize the telomeric restriction fragments (TRFs). Mean TRF length (MTL) and standard deviation were calculated using TeloTool (Gohring et al., 2014).

### 4.3 RNA sample preparation and RNA sequencing

Total RNA was isolated using Qiazol Lysis Reagent (Qiagen) and purified using the MiRNeasy system (Qiagen). RNA purity (260/230 and 280/260 OD ratios) was measured using Nanodrop Spectrophotometer (Nanodrop 1000). RNA concentration was measured using Fluorometer (Thermo Scientific), and RNA integrity was evaluated using the Agilent 4200 TapeStation system (Agilent Technologies). RNA‐seq was carried out by Novogene using the Illumina NovaSeq 6000 Sequencing System.

### 4.4 Real‐time PCR (RT‐qPCR)

One μg of total RNA was used to synthesize the corresponding cDNA using the GoScript™ Reverse Transcriptase Kit (Promega). RT‐qPCR was performed using the Power SYBR™ Green PCR Master Mix (Thermo) and the StepOne RT‐qPCR System (Thermo). Primers are listed in Table 1. The reactions were carried out in triplicates and averaged, and computaional analysis was done using the delta–delta Ct formula with the *GAPDH* gene as normalizing control.

**TABLE 1:** RT‐PCR primers.

Gene	Orientation sequence	
OAS2	Forward	GCTTCCGACAATCAACAGCCAAG
	Reverse	CTTGACGATTTTGTGCCGCTCG
STAT1	Forward	ATGGCAGTCTGGCGGCTGAATT
	Reverse	CCAAACCAGGCTGGCACAATTG
MX1	Forward	GGCTGTTTACCAGACTCCGACA
	Reverse	CACAAAGCCTGGCAGCTCTCTA
ISG15	Forward	AGATCACCCAGAAGATCG
	Reverse	TGTTATTCCTCACCAGGATG
FBN2	Forward	CCGAAGGTTTCACTGGTGATGG
	Reverse	CCATCTCACACTCGCAGCGATA
RPELP	Forward	CGCCATCAACAACAGGCTGGAA
	Reverse	CGCCATCAACAACAGGCTGGAA
VCAM1	Forward	GATTCTGTGCCCACAGTAAGGC
	Reverse	TGGTCACAGAGCCACCTTCTTG
ANKRD1	Forward	CGACTCCTGATTATGTATGGCGC
	Reverse	GCTTTGGTTCCATTCTGCCAGTG
LIF	Forward	AGATCAGGAGCCAACTGGCACA
	Reverse	GCCACATAGCTTGTCCAGGTTG
SERPINE1	Forward	CTCATCAGCCACTGGAAAGGCA
	Reverse	GACTCGTGAAGTCAGCCTGAAAC
TGFB2	Forward	AAGAAGCGTGCTTTGGATGCGG
	Reverse	ATGCTCCAGCACAGAAGTTGGC
CCN2	Forward	CTTGCGAAGCTGACCTGGAAGA
	Reverse	CCGTCGGTACATACTCCACAGA
GAPDH	Forward	TTGGCTACAGCAACAGGGTG
	Reverse	GGGGAGATTCAGTGTGGTGG

### 4.5 Quality control and read processing

Quality control checks on the raw sequencing data (FASTQ files) were performed using FastQC v0.11.9 (Andrews, 2010). Adaptor clipping and leading and trailing low quality or N bases trimming was done using Trimmomatic v0.36 (Bolger et al., 2014) in paired‐end mode. Additional quality control checks were done after trimming, using the same FastQC program.

### 4.6 Alignment and gene expression

Sequenced reads were aligned to the human genome (hg19) using Rsubread v2.0.0 (Liao et al., 2019) ‘align’ function (sortReadsByCoordinates = True, detectSV = False) in paired‐end mode, with the human UCSC refseq genes (hg19) GTF as an external annotation file (Speir et al., 2016). Alignment statistics were examined using Rsubread's ‘propmapped’ function and Picard v2.5.0 (“Picard Toolkit” 2019) ‘CollectRnaSeqMetrics’. Bam files of each sample that was sequenced on several lanes were merged at this stage using the Samtools v1.3.1 (Danecek et al., 2021) ‘merge’ command. Gene expression count matrix was calculated using Rsubread ‘featureCounts’ (countMultiMappingReads = False) in paired end mode to count the number of reads that map to each gene, with the GENCODE GRCh37 (hg19) v25 GTF as an external annotation file (Harrow et al., 2012). The reads were then normalized by TPM (Transcripts per Kilobase Million) using the total exonic length acquired from the same GENCODE database. In order to remain with only genes that were robustly expressed in the samples, we continued only with genes that had a value of at least 1.0 TPM in all replicates of at least one biological condition in the dataset.

### 4.7 PCA and hierarchical clustering

PCA and hierarchical clustering were done using the R v3.5.3 ‘stats’ package. Visualization was done through the ‘ggthemes’ and ‘factoextra’ packages.

### 4.8 Differential expression analysis

Differential gene expression was calculated using DESeq2 (Love et al., 2014) package v1.26.0, using the thresholds: adjusted *p* < 0.05 and absolute log_2_ fold change >1.5. This was done for 10 comparisons throughout the dataset (Table 1). In order to combat the high variability between the different human samples and further increase the veracity of the resulting genes, an additional step was taken for each comparison called ‘separation tests’. For each gene that passed our DESeq2 thresholds, we verified that the respective differential expression was consistent throughout all replicates (minimum log_2_ fold change of >1.2). If one or more replicate samples did not show this trend, the gene was removed from the respective gene set. The number of remaining genes and percentage of removed genes are shown in Table 1. Finally, a union of the genes that passed all stages up to this point was created, to be used in the next step of the analysis.

### 4.9 Cluster and enrichment analysis

The final gene set of differentially expressed genes (DEGs) underwent cluster analysis using EXPANDER v8.0 (Hait et al., 2019) and the integrated CLICK algorithm (Sharan et al., 2003), in order to group together genes that show similar behaviors over a set of conditions. The full list of clusters can be viewed in Table S3. GO functional enrichment analysis of the clusters was performed using clusterProfiler v3.14.3 (Yu et al., 2012) and the Bioconductor genome wide annotation for human v3.10.0 (Carlson, 2019). The background genes were the full list of expressed genes in the dataset, the threshold was adjusted *p* < 0.05, calculated using Benjamini & Hochberg correction. Heatmap visualization was done using Heatmapper (Babicki et al., 2016).

### 4.10 Gene set enrichment analysis (GSEA)

Gene set enrichment analysis (Subramanian et al., 2005) was also performed using clusterProfiler v3.14.3 (Yu et al., 2012), with the Molecular Signatures Database (MSigDB) (Liberzon et al., 2011) Hallmark gene set v7.2 (Liberzon et al., 2015). This was performed on the entire set of expressed protein‐coding genes; log_2_ fold change values were calculated manually adding ε=0.5 to the raw TPM values (to avoid FC inflation for lowly expressed genes), and once again a threshold of adjusted *p* < 0.05 using Benjamini & Hochberg correction. The comparisons are detailed in the respective GSEA table and plots. Visualization was done with the help of the R package ‘enrichplot’ v1.6.1 (Yu, 2019).

### 4.11 PPI network visualization of selected genes

The PPI networks were built using the Search Tool for the Retrieval of Interacting Genes/Proteins (*STRING*) (Huang et al., 2018). The PPI network was then visualized using Cytoscape software v3.9.1 (Shannon et al., 2003). Nodes were colored based on the relevant log_2_ fold change. Next, the molecular complexes were extracted by isolating the densest part of these networks using MCODE app v2.0.0 (Bader & Hogue, 2003), under default settings. Enrichment of the MCODE subnetworks was done using the stringApp enrichment tool from Cytoscape (Doncheva et al., 2019).

### 4.12 Public expression data analysis from gene expression omnibus

The microarray expression data (GSE35347) (Nayler et al., 2012) was obtained from NCBI Gene Expression Omnibus using the GEOquery R package v2.40.0 (Davis & Meltzer, 2007). Only primary skin fibroblasts samples, isolated from Ctrl and A‐T patients were used (Ctrl: GSM866594, GSM866595, GSM866596 A‐T: GSM866606, GSM866607, and GSM866608). Differentially Expressed Genes were defined using the “Limma” R package v.3.26.8 (Ritchie et al., 2015). Ranked genes were analyzed using the Gene Set Enrichment Analysis (GSEA; v1.58.0) (Subramanian et al., 2005) with the molecular signatures database (MSigDB v7.5.1) gene sets (h: hallmark gene sets). GSEA graph was generated using ClusterProfiler v3.15 (Yu et al., 2012).

## AUTHOR CONTRIBUTIONS

Majd Haj was involved in conceptualization, experimental design and work, writing the initial draft. Amit Levon was involved in bioinformatic analysis—design and carrying out, writing the manuscript. Yann Frey was involved in bioinformatic analysis—design and carrying out, data mining, writing the manuscript. Noa Hourvitz was involved in experimental design and work. Yehuda Tzfati was involved in conceptualization, guidance and supervision. Judith Campisi was involved in conceptualization, guidance and supervision of experimental work, discussion of the data. Ran Elkon was involved in bioinformatic analysis—conceptualization, guidance and supervision, writing the manuscript. Yael Ziv was involved in conceptualization, experimental work, guidance and supervision of experimental work, writing the manuscript. Yosef Shiloh was involved in conceptualization, guidance and supervision of experimental work and bioinformatic analysis, writing the manuscript, funding.

## CONFLICT OF INTEREST STATEMENT

The authors declare no conflict‐of‐interests.

## Supporting information


**Figure S1.** **Figure S2**. **Figure S3**. **Figure S4**. **Figure S5**. **Figure S6**. **Figure S7**. **Figure S8**.

**Table S1.** **Table S2**. **Table S3**.

## Data Availability

Raw data for all 33 samples and initial data processing can be found on the GEO website under accession number GSE182410.

## References

[acel13869-bib-0001] Aguado, J. , Chaggar, H. K. , Gomez‐Inclan, C. , Shaker, M. R. , Leeson, H. C. , Mackay‐Sim, A. , & Wolvetang, E. J. (2021a). Inhibition of the cGAS‐STING pathway ameliorates the premature senescence hallmarks of ataxia‐telangiectasia brain organoids. Aging Cell, 20(9), e13468. 10.1111/acel.13468 34459078 PMC8441292

[acel13869-bib-0002] Aguado, J. , Gomez‐Inclan, C. , Leeson, H. C. , Lavin, M. F. , Shiloh, Y. , & Wolvetang, E. J. (2022). The hallmarks of aging in ataxia‐telangiectasia. Ageing Research Reviews, 79, 101653. 10.1016/j.arr.2022.101653 35644374

[acel13869-bib-0003] Andrews S . (2010). FastQC: A quality control tool for high throughput sequence data [Online]. Retrieved from http://www.bioinformatics.babraham.ac.uk/projects/fastqc/.

[acel13869-bib-0004] Babicki, S. , Arndt, D. , Marcu, A. , Liang, Y. , Grant, J. R. , Maciejewski, A. , & Wishart, D. S. (2016). Heatmapper: Web‐enabled heat mapping for all. Nucleic Acids Research, 44(W1), W147–W153. 10.1093/nar/gkw419 27190236 PMC4987948

[acel13869-bib-0005] Bader, G. D. , & Hogue, C. W. V. (2003). An automated method for finding molecular complexes in large protein interaction networks. BMC Bioinformatics, 4(1), 2. 10.1186/1471-2105-4-2 12525261 PMC149346

[acel13869-bib-0006] Bakhoum, S. F. , Ngo, B. , Laughney, A. M. , Cavallo, J. A. , Murphy, C. J. , Ly, P. , & Cantley, L. C. (2018). Chromosomal instability drives metastasis through a cytosolic DNA response. Nature, 553(7689), 467–472. 10.1038/nature25432 29342134 PMC5785464

[acel13869-bib-0007] Bakkenist, C. J. , & Kastan, M. B. (2003). DNA damage activates ATM through intermolecular autophosphorylation and dimer dissociation. Nature, 421(6922), 499–506. 10.1038/nature01368 12556884

[acel13869-bib-0008] Birch, J. , & Gil, J. (2020). Senescence and the SASP: Many therapeutic avenues. Genes & Development, 34(23–24), 1565–1576. 10.1101/gad.343129.120 33262144 PMC7706700

[acel13869-bib-0009] Blokland, K. E. C. , Pouwels, S. D. , Schuliga, M. , Knight, D. A. , & Burgess, J. K. (2020). Regulation of cellular senescence by extracellular matrix during chronic fibrotic diseases. Clinical Science (London, England), 134(20), 2681–2706. 10.1042/CS20190893 PMC7578566 33084883

[acel13869-bib-0010] Bolger, A. M. , Lohse, M. , & Usadel, B. (2014). Trimmomatic: A flexible trimmer for Illumina sequence data. Bioinformatics, 30(15), 2114–2120. 10.1093/bioinformatics/btu170 24695404 PMC4103590

[acel13869-bib-0011] Borden, E. C. , Sen, G. C. , Uze, G. , Silverman, R. H. , Ransohoff, R. M. , Foster, G. R. , & Stark, G. R. (2007). Interferons at age 50: Past, current and future impact on biomedicine. Nature Reviews. Drug Discovery, 6(12), 975–990. 10.1038/nrd2422 18049472 PMC7097588

[acel13869-bib-0012] Carlson, M. (2019). org.Hs.eg.db: Genome wide annotation for human: R package version 3.10.0.

[acel13869-bib-0013] Chatterjee, N. , & Walker, G. C. (2017). Mechanisms of DNA damage, repair, and mutagenesis. Environmental and Molecular Mutagenesis, 58, 235–263. 10.1002/em.22087 28485537 PMC5474181

[acel13869-bib-0014] Chen, Q. , Fischer, A. , Reagan, J. D. , Yan, L. J. , & Ames, B. N. (1995). Oxidative DNA damage and senescence of human diploid fibroblast cells. Proceedings of the National Academy of Sciences of the United States of America, 92(10), 4337–4341. 10.1073/pnas.92.10.4337 7753808 PMC41939

[acel13869-bib-0015] Coppe, J. P. , Desprez, P. Y. , Krtolica, A. , & Campisi, J. (2010). The senescence‐associated secretory phenotype: The dark side of tumor suppression. Annual Review of Pathology, 5, 99–118. 10.1146/annurev-pathol-121808-102144 PMC4166495 20078217

[acel13869-bib-0016] Danecek, P. , Bonfield, J. K. , Liddle, J. , Marshall, J. , Ohan, V. , Pollard, M. O. , & Li, H. (2021). Twelve years of SAMtools and BCFtools. GigaScience, 10(2), giab008. 10.1093/gigascience/giab008 33590861 PMC7931819

[acel13869-bib-0017] Davis, S. , & Meltzer, P. S. (2007). GEOquery: A bridge between the gene expression omnibus (GEO) and BioConductor. Bioinformatics, 23(14), 1846–1847. 10.1093/bioinformatics/btm254 17496320

[acel13869-bib-0018] Davis, T. , & Kipling, D. (2009). Assessing the role of stress signalling via p38 MAP kinase in the premature senescence of ataxia telangiectasia and Werner syndrome fibroblasts. Biogerontology, 10(3), 253–266. 10.1007/s10522-008-9179-x 18830681

[acel13869-bib-0019] De Blander, H. , Morel, A. P. , Senaratne, A. P. , Ouzounova, M. , & Puisieux, A. (2021). Cellular plasticity: A route to senescence exit and tumorigenesis. Cancers (Basel), 13(18). 10.3390/cancers13184561 PMC8468602 34572787

[acel13869-bib-0020] Di Micco, R. , Krizhanovsky, V. , Baker, D. , & d'Adda di Fagagna, F. (2021). Cellular senescence in ageing: From mechanisms to therapeutic opportunities. Nature Reviews. Molecular Cell Biology, 22(2), 75–95. 10.1038/s41580-020-00314-w 33328614 PMC8344376

[acel13869-bib-0021] Doncheva, N. T. , Morris, J. H. , Gorodkin, J. , & Jensen, L. J. (2019). Cytoscape StringApp: Network analysis and visualization of proteomics data. Journal of Proteome Research, 18(2), 623–632. 10.1021/acs.jproteome.8b00702 30450911 PMC6800166

[acel13869-bib-0022] Dou, Z. , Ghosh, K. , Vizioli, M. G. , Zhu, J. , Sen, P. , Wangensteen, K. J. , & Berger, S. L. (2017). Cytoplasmic chromatin triggers inflammation in senescence and cancer. Nature, 550(7676), 402–406. 10.1038/nature24050 28976970 PMC5850938

[acel13869-bib-0023] Elmore, E. , & Swift, M. (1976). Growth of cultured cells from patients with ataxia‐telangiectasia. Journal of Cellular Physiology, 8(3), 429–431. 10.1002/jcp.1040890308 977661

[acel13869-bib-0024] Frisch, S. M. , & MacFawn, I. P. (2020). Type I interferons and related pathways in cell senescence. Aging Cell, 19(10), e13234. 10.1111/acel.13234 32918364 PMC7576263

[acel13869-bib-0025] Gilad, S. , Khosravi, R. , Shkedy, D. , Uziel, T. , Ziv, Y. , Savitsky, K. , & Bar‐Shira, A. (1996). Predominance of null mutations in ataxia‐telangiectasia. Human Molecular Genetics, 5(4), 433–439. 8845835 10.1093/hmg/5.4.433

[acel13869-bib-0026] Gohring, J. , Fulcher, N. , Jacak, J. , & Riha, K. (2014). TeloTool: A new tool for telomere length measurement from terminal restriction fragment analysis with improved probe intensity correction. Nucleic Acids Research, 42(3), e21. 10.1093/nar/gkt1315 24366880 PMC3919618

[acel13869-bib-0027] Goldstein, M. , & Kastan, M. B. (2015). The DNA damage response: Implications for tumor responses to radiation and chemotherapy. Annual Review of Medicine, 66, 129–143. 10.1146/annurev-med-081313-121208 25423595

[acel13869-bib-0028] Guo, Z. , Kozlov, S. , Lavin, M. F. , Person, M. D. , & Paull, T. T. (2010). ATM activation by oxidative stress. Science, 330(6003), 517–521. 10.1126/science.1192912 20966255

[acel13869-bib-0029] Haag, S. M. , Gulen, M. F. , Reymond, L. , Gibelin, A. , Abrami, L. , Decout, A. , & Ablasser, A. (2018). Targeting STING with covalent small‐molecule inhibitors. Nature, 559(7713), 269–273. 10.1038/s41586-018-0287-8 29973723

[acel13869-bib-0030] Hait, T. A. , Maron‐Katz, A. , Sagir, D. , Amar, D. , Ulitsky, I. , Linhart, C. , & Shamir, R. (2019). The EXPANDER integrated platform for transcriptome analysis. Journal of Molecular Biology, 431(13), 2398–2406. 10.1016/j.jmb.2019.05.013 31100387

[acel13869-bib-0031] Han, X. , Lei, Q. , Xie, J. , Liu, H. , Sun, H. , Jing, L. , & Gou, X. (2022). Potential regulators of the senescence‐associated secretory phenotype during senescence and ageing. The Journals of Gerontology. Series A: Biological Sciences and Medical Sciences, 77, 2207–2218. 10.1093/gerona/glac097 35524726

[acel13869-bib-0032] Harding, S. M. , Benci, J. L. , Irianto, J. , Discher, D. E. , Minn, A. J. , & Greenberg, R. A. (2017). Mitotic progression following DNA damage enables pattern recognition within micronuclei. Nature, 548(7668), 466–470. 10.1038/nature23470 28759889 PMC5857357

[acel13869-bib-0033] Harrow, J. , Frankish, A. , Gonzalez, J. M. , Tapanari, E. , Diekhans, M. , Kokocinski, F. , Aken, B. L. , Barrell, D. , Zadissa, A. , Searle, S. , Barnes, I. , Bignell, A. , Boychenko, V. , Hunt, T. , Kay, M. , Mukherjee, G. , Rajan, J. , Despacio‐Reyes, G. , Saunders, G. … Hubbard, T. J. (2012). GENCODE: The reference human genome annotation for the ENCODE project. Genome Research, 22(9), 1760–1774. 10.1101/gr.135350.111 22955987 PMC3431492

[acel13869-bib-0034] Hayflick, L. (1998). A brief history of the mortality and immortality of cultured cells. The Keio Journal of Medicine, 47(3), 174–182. 10.2302/kjm.47.174 9785764

[acel13869-bib-0035] Hernandez‐Segura, A. , de Jong, T. V. , Melov, S. , Guryev, V. , Campisi, J. , & Demaria, M. (2017). Unmasking transcriptional heterogeneity in senescent cells. Current Biology, 27(17), 2652–2660 e2654. 10.1016/j.cub.2017.07.033 28844647 PMC5788810

[acel13869-bib-0036] Hernandez‐Segura, A. , Nehme, J. , & Demaria, M. (2018). Hallmarks of cellular senescence. Trends in Cell Biology, 28(6), 436–453. 10.1016/j.tcb.2018.02.001 29477613

[acel13869-bib-0037] Hoar, D. I. (1975). Letter: Phenotypic manifestations of ataxia‐telangiectasia. Lancet, 2(7943), 1048. 10.1016/s0140-6736(75)90347-5 53540

[acel13869-bib-0038] Huang, J. K. , Carlin, D. E. , Yu, M. K. , Zhang, W. , Kreisberg, J. F. , Tamayo, P. , & Ideker, T. (2018). Systematic evaluation of molecular networks for discovery of disease genes. Cell Systems, 6(4), 484–495.e485. 10.1016/j.cels.2018.03.001 29605183 PMC5920724

[acel13869-bib-0039] Jachimowicz, R. D. , Beleggia, F. , Isensee, J. , Velpula, B. B. , Goergens, J. , Bustos, M. A. , & Shiloh, Y. (2019). UBQLN4 represses homologous recombination and is overexpressed in aggressive tumors. Cell, 176(3), 505–519 e522. 10.1016/j.cell.2018.11.024 30612738

[acel13869-bib-0040] Keeley, T. P. , & Mann, G. E. (2019). Defining physiological normoxia for improved translation of cell physiology to animal models and humans. Physiological Reviews, 99(1), 161–234. 10.1152/physrev.00041.2017 30354965

[acel13869-bib-0041] Kreienkamp, R. , Graziano, S. , Coll‐Bonfill, N. , Bedia‐Diaz, G. , Cybulla, E. , Vindigni, A. , & Gonzalo, S. (2018). A cell‐intrinsic interferon‐like response links replication stress to cellular aging caused by Progerin. Cell Reports, 22(8), 2006–2015. 10.1016/j.celrep.2018.01.090 29466729 PMC5848491

[acel13869-bib-0042] Lama, L. , Adura, C. , Xie, W. , Tomita, D. , Kamei, T. , Kuryavyi, V. , & Tuschl, T. (2019). Development of human cGAS‐specific small‐molecule inhibitors for repression of dsDNA‐triggered interferon expression. Nature Communications, 10(1), 2261. 10.1038/s41467-019-08620-4 PMC6529454 31113940

[acel13869-bib-0043] Lamm, N. , Ordan, E. , Shponkin, R. , Richler, C. , Aker, M. , & Tzfati, Y. (2009). Diminished telomeric 3′ overhangs are associated with telomere dysfunction in Hoyeraal‐Hreidarsson syndrome. PLoS One, 4(5), e5666. 10.1371/journal.pone.0005666 19461895 PMC2680952

[acel13869-bib-0044] Lee, J. H. , & Paull, T. T. (2021). Cellular functions of the protein kinase ATM and their relevance to human disease. Nature Reviews. Molecular Cell Biology, 22(12), 796–814. 10.1038/s41580-021-00394-2 34429537

[acel13869-bib-0045] Levi, N. , Papismadov, N. , Solomonov, I. , Sagi, I. , & Krizhanovsky, V. (2020). The ECM path of senescence in aging: Components and modifiers. The FEBS Journal, 287(13), 2636–2646. 10.1111/febs.15282 32145148

[acel13869-bib-0046] Liao, Y. , Smyth, G. K. , & Shi, W. (2019). The R package Rsubread is easier, faster, cheaper and better for alignment and quantification of RNA sequencing reads. Nucleic Acids Research, 47(8), e47. 10.1093/nar/gkz114 30783653 PMC6486549

[acel13869-bib-0047] Liberzon, A. , Birger, C. , Thorvaldsdóttir, H. , Ghandi, M. , Mesirov, J. P. , & Tamayo, P. (2015). The molecular signatures database (MSigDB) hallmark gene set collection. Cell Systems, 1(6), 417–425. 10.1016/j.cels.2015.12.004 26771021 PMC4707969

[acel13869-bib-0048] Liberzon, A. , Subramanian, A. , Pinchback, R. , Thorvaldsdóttir, H. , Tamayo, P. , & Mesirov, J. P. (2011). Molecular signatures database (MSigDB) 3.0. Bioinformatics, 27(12), 1739–1740. 10.1093/bioinformatics/btr260 21546393 PMC3106198

[acel13869-bib-0049] Lopes‐Paciencia, S. , Saint‐Germain, E. , Rowell, M. C. , Ruiz, A. F. , Kalegari, P. , & Ferbeyre, G. (2019). The senescence‐associated secretory phenotype and its regulation. Cytokine, 117, 15–22. 10.1016/j.cyto.2019.01.013 30776684

[acel13869-bib-0050] Love, M. I. , Huber, W. , & Anders, S. (2014). Moderated estimation of fold change and dispersion for RNA‐seq data with DESeq2. Genome Biology, 15(12), 550. 10.1186/s13059-014-0550-8 25516281 PMC4302049

[acel13869-bib-0051] Lovisa, S. (2021). Epithelial‐to‐mesenchymal transition in fibrosis: Concepts and targeting strategies. Frontiers in Pharmacology, 12, 737570. 10.3389/fphar.2021.737570 34557100 PMC8454779

[acel13869-bib-0052] MacDonald, K. M. , Nicholson‐Puthenveedu, S. , Tageldein, M. M. , Khasnis, S. , Arrowsmith, C. H. , & Harding, S. M. (2023). Antecedent chromatin organization determines cGAS recruitment to ruptured micronuclei. Nature Communications, 14(1), 556. 10.1038/s41467-023-36195-8 PMC9894866 36732527

[acel13869-bib-0053] Metcalfe, J. A. , Parkhill, J. , Campbell, L. , Stacey, M. , Biggs, P. , Byrd, P. J. , & Taylor, A. M. (1996). Accelerated telomere shortening in ataxia telangiectasia. Nature Genetics, 13(3), 350–353. 10.1038/ng0796-350 8673136

[acel13869-bib-0054] Miar, A. , Arnaiz, E. , Bridges, E. , Beedie, S. , Cribbs, A. P. , Downes, D. J. , & Harris, A. L. (2020). Hypoxia induces transcriptional and translational downregulation of the type I IFN pathway in multiple cancer cell types. Cancer Research, 80(23), 5245–5256. 10.1158/0008-5472.CAN-19-2306 33115807 PMC7611234

[acel13869-bib-0055] Mullani, N. , Porozhan, Y. , Mangelinck, A. , Rachez, C. , Costallat, M. , Batsche, E. , & Muchardt, C. (2021). Reduced RNA turnover as a driver of cellular senescence. Life Science Alliance, 4(3), e202000809. 10.26508/lsa.202000809 33446491 PMC7812316

[acel13869-bib-0056] Nayler, S. , Gatei, M. , Kozlov, S. , Gatti, R. , Mar, J. C. , Wells, C. A. , & Wolvetang, E. (2012). Induced pluripotent stem cells from ataxia‐telangiectasia recapitulate the cellular phenotype. Stem Cells Translational Medicine, 1(7), 523–535. 10.5966/sctm.2012-0024 23197857 PMC3659724

[acel13869-bib-0057] Pandita, T. K. (2001). The role of ATM in telomere structure and function. Radiation Research, 156(5 Pt 2), 642–647. 11604086 10.1667/0033-7587(2001)156[0642:troait]2.0.co;2

[acel13869-bib-0058] Parrinello, S. , Samper, E. , Krtolica, A. , Goldstein, J. , Melov, S. , & Campisi, J. (2003). Oxygen sensitivity severely limits the replicative lifespan of murine fibroblasts. Nature Cell Biology, 5(8), 741–747. 10.1038/ncb1024 12855956 PMC4940195

[acel13869-bib-0059] Ritchie, C. , Carozza, J. A. , & Li, L. (2022). Biochemistry, cell biology, and pathophysiology of the innate immune cGAS‐cGAMP‐STING pathway. Annual Review of Biochemistry, 91, 599–628. 10.1146/annurev-biochem-040320-101629 35287475

[acel13869-bib-0060] Ritchie, M. E. , Phipson, B. , Wu, D. , Hu, Y. , Law, C. W. , Shi, W. , & Smyth, G. K. (2015). Limma powers differential expression analyses for RNA‐sequencing and microarray studies. Nucleic Acids Research, 43(7), e47. 10.1093/nar/gkv007 25605792 PMC4402510

[acel13869-bib-0061] Roger, L. , Tomas, F. , & Gire, V. (2021). Mechanisms and regulation of cellular senescence. International Journal of Molecular Sciences, 22(23). 10.3390/ijms222313173 PMC8658264 34884978

[acel13869-bib-0062] Rothblum‐Oviatt, C. , Wright, J. , Lefton‐Greif, M. A. , McGrath‐Morrow, S. A. , Crawford, T. O. , & Lederman, H. M. (2016). Ataxia telangiectasia: A review. Orphanet Journal of Rare Diseases, 11(1), 159. 10.1186/s13023-016-0543-7 27884168 PMC5123280

[acel13869-bib-0063] Savitsky, K. , Bar‐Shira, A. , Gilad, S. , Rotman, G. , Ziv, Y. , Vanagaite, L. , Tagle, D.A. , Smith, S. , Uziel, T. , Sfez, S. , Ashkenazi, M. , Pecker, I. , Frydman, M. , Harnik, R. , Patanjali, S.R. , Simmons, A. , Clines, G.A. , Sartiel, A. , Gatti, R.A. … Shiloh, Y. (1995). A single ataxia telangiectasia gene with a product similar to PI‐3 kinase. Science, 268(5218), 1749–1753. 10.1126/science.7792600 7792600

[acel13869-bib-0064] Seno, K. , Tanikawa, N. , Takahashi, H. , Ohkuchi, A. , Suzuki, H. , Matsubara, S. , & Shirasuna, K. (2018). Oxygen concentration modulates cellular senescence and autophagy in human trophoblast cells. American Journal of Reproductive Immunology, 79(6), e12826. 10.1111/aji.12826 29446169

[acel13869-bib-0065] Shannon, P. , Markiel, A. , Ozier, O. , Baliga, N. S. , Wang, J. T. , Ramage, D. , & Ideker, T. (2003). Cytoscape: A software environment for integrated models of biomolecular interaction networks. Genome Research, 13(11), 2498–2504. 10.1101/gr.1239303 14597658 PMC403769

[acel13869-bib-0066] Sharan, R. , Maron‐Katz, A. , & Shamir, R. (2003). CLICK and EXPANDER: A system for clustering and visualizing gene expression data. Bioinformatics, 19(14), 1787–1799. 10.1093/bioinformatics/btg232 14512350

[acel13869-bib-0067] Sheekey, E. , & Narita, M. (2021). p53 in senescence–it's a marathon not a sprint. FEBS Journal, 290, 1212–1220. 10.1111/febs.16325 34921507

[acel13869-bib-0068] Shibata, A. , & Jeggo, P. A. (2021). ATM's role in the repair of DNA double‐strand breaks. Genes (Basel), 12(9). 10.3390/genes12091370 PMC8466060 34573351

[acel13869-bib-0069] Shiloh, Y. , & Lederman, H. M. (2017). Ataxia‐telangiectasia (A‐T): An emerging dimension of premature ageing. Ageing Research Review, 33, 76–88. 10.1016/j.arr.2016.05.002 27181190

[acel13869-bib-0070] Shiloh, Y. , Tabor, E. , & Becker, Y. (1982). Colony‐forming ability of ataxia‐telangiectasia skin fibroblasts is an indicator of their early senescence and increased demand for growth factors. Experimental Cell Research, 140(1), 191–199. 10.1016/0014-4827(82)90169-0 6213420

[acel13869-bib-0071] Shmulevich, R. , & Krizhanovsky, V. (2021). Cell senescence, DNA damage, and metabolism. Antioxidants & Redox Signaling, 34(4), 324–334. 10.1089/ars.2020.8043 32212823

[acel13869-bib-0072] Speir, M. L. , Zweig, A. S. , Rosenbloom, K. R. , Raney, B. J. , Paten, B. , Nejad, P. , & Kent, W. J. (2016). The UCSC genome browser database: 2016 update. Nucleic Acids Research, 44(D1), D717–D725. 10.1093/nar/gkv1275 26590259 PMC4702902

[acel13869-bib-0073] Sturmlechner, I. , Zhang, C. , Sine, C. C. , van Deursen, E. J. , Jeganathan, K. B. , Hamada, N. , & van Deursen, J. M. (2021). p21 produces a bioactive secretome that places stressed cells under immunosurveillance. Science, 374(6567), eabb3420. 10.1126/science.abb3420 34709885 PMC8985214

[acel13869-bib-0074] Subramanian, A. , Tamayo, P. , Mootha, V. K. , Mukherjee, S. , Ebert, B. L. , Gillette, M. A. , & Mesirov, J. P. (2005a). Gene set enrichment analysis: A knowledge‐based approach for interpreting genome‐wide expression profiles. Proceedings of the National Academy of Sciences, 102(43), 15545–15550. 10.1073/pnas.0506580102 PMC1239896 16199517

[acel13869-bib-0075] Taylor, A. M. R. , Rothblum‐Oviatt, C. , Ellis, N. A. , Hickson, I. D. , Meyer, S. , Crawford, T. O. , & Stewart, G. S. (2019). Chromosome instability syndromes. Nature Reviews Disease Primers, 5(1), 64. 10.1038/s41572-019-0113-0 PMC10617425 31537806

[acel13869-bib-0076] Picard Toolkit . (2019). Broad Institute, GitHub Repository. Retrieved from http://broadinstitute.github.io/picard/.

[acel13869-bib-0077] Tubbs, A. , & Nussenzweig, A. (2017). Endogenous DNA damage as a source of genomic instability in cancer. Cell, 168(4), 644–656. 10.1016/j.cell.2017.01.002 28187286 PMC6591730

[acel13869-bib-0078] Vaddavalli, P. L. , & Schumacher, B. (2022). The p53 network: Cellular and systemic DNA damage responses in cancer and aging. Trends in Genetics, 38(6), 598–612. 10.1016/j.tig.2022.02.010 35346511

[acel13869-bib-0079] van Vliet, T. , Varela‐Eirin, M. , Wang, B. , Borghesan, M. , Brandenburg, S. M. , Franzin, R. , & Demaria, M. (2021). Physiological hypoxia restrains the senescence‐associated secretory phenotype via AMPK‐mediated mTOR suppression. Molecular Cell, 81(9), 2041–2052. e2046. 10.1016/j.molcel.2021.03.018 33823141

[acel13869-bib-0080] Wiley, C. D. , & Campisi, J. (2021). The metabolic roots of senescence: Mechanisms and opportunities for intervention. Nature Metabolism, 3(10), 1290–1301. 10.1038/s42255-021-00483-8 PMC8889622 34663974

[acel13869-bib-0081] Yousefzadeh, M. , Henpita, C. , Vyas, R. , Soto‐Palma, C. , Robbins, P. , & Niedernhofer, L. (2021). DNA damage‐how and why we age? eLife, 10. 10.7554/eLife.62852 PMC7846274 33512317

[acel13869-bib-0082] Yu, G. (2019). enrichplot: visualization of functional enrichment result. R package version 1.6.1. Retrieved from https://github.com/GuangchuangYu/enrichplot

[acel13869-bib-0083] Yu, G. , Wang, L. G. , Han, Y. , & He, Q. Y. (2012). clusterProfiler: An R package for comparing biological themes among gene clusters. Omics, 16(5), 284–287. 10.1089/omi.2011.0118 22455463 PMC3339379

