# Supplementary material for: Accelerated replicative senescence of ataxia‐telangiectasia skin fibroblasts is retained at physiologic oxygen levels, with unique and common transcriptional patterns
Source: Aging Cell. 2023 May 30;22(8):e13869. doi: 10.1111/acel.13869 (PMC10410012; doi:10.1111/acel.13869)
Supplement: Supplementary file 1 — Figure S1. Figure S2. Figure S3. Figure S4. Figure S5. Figure S6. Figure S7. Figure S8. [file ACEL-22-e13869-s001.pptx]

## Slide 1
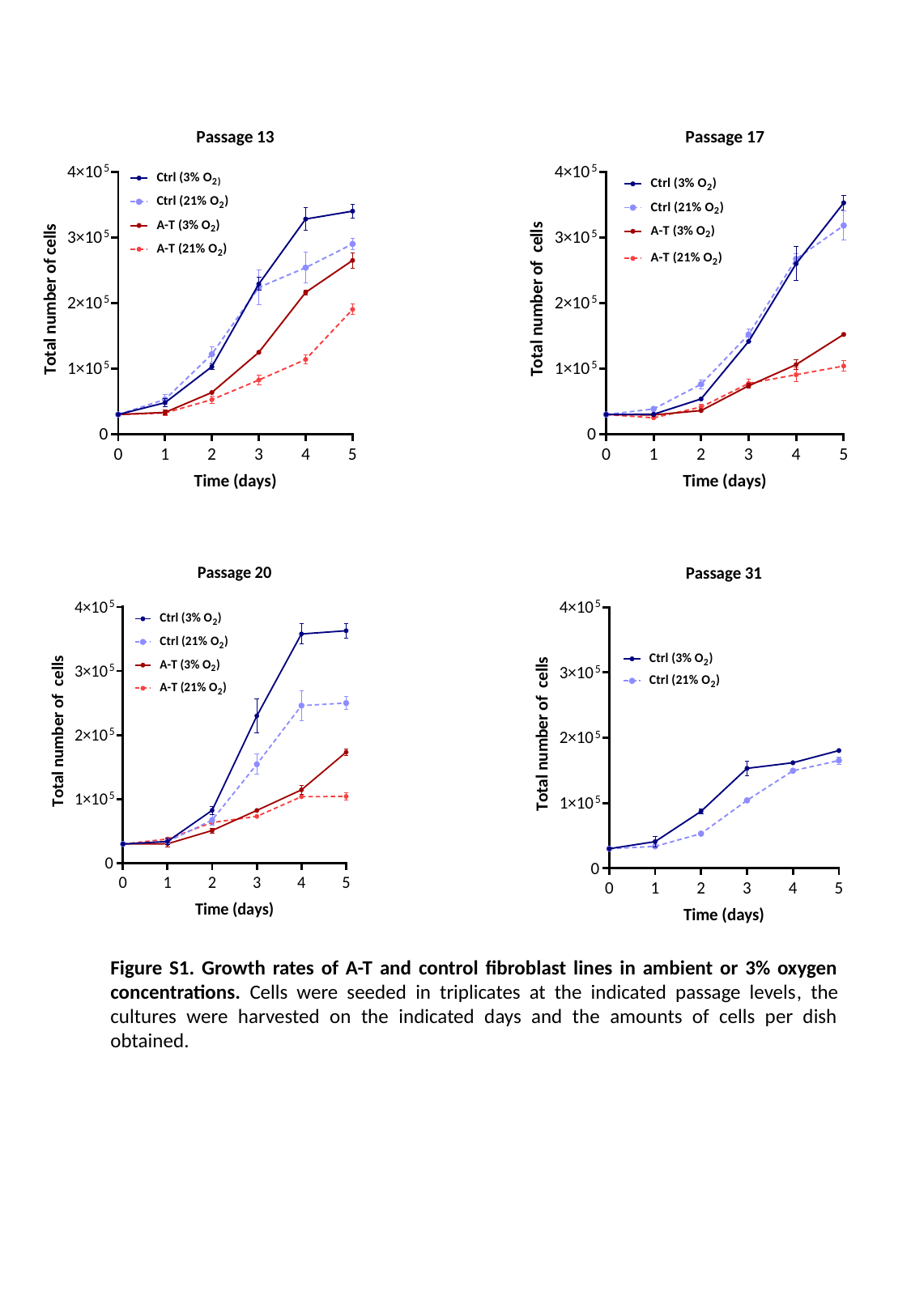

Figure S1. Growth rates of A-T and control fibroblast lines in ambient or 3% oxygen concentrations. Cells were seeded in triplicates at the indicated passage levels, the cultures were harvested on the indicated days and the amounts of cells per dish obtained.

## Slide 2
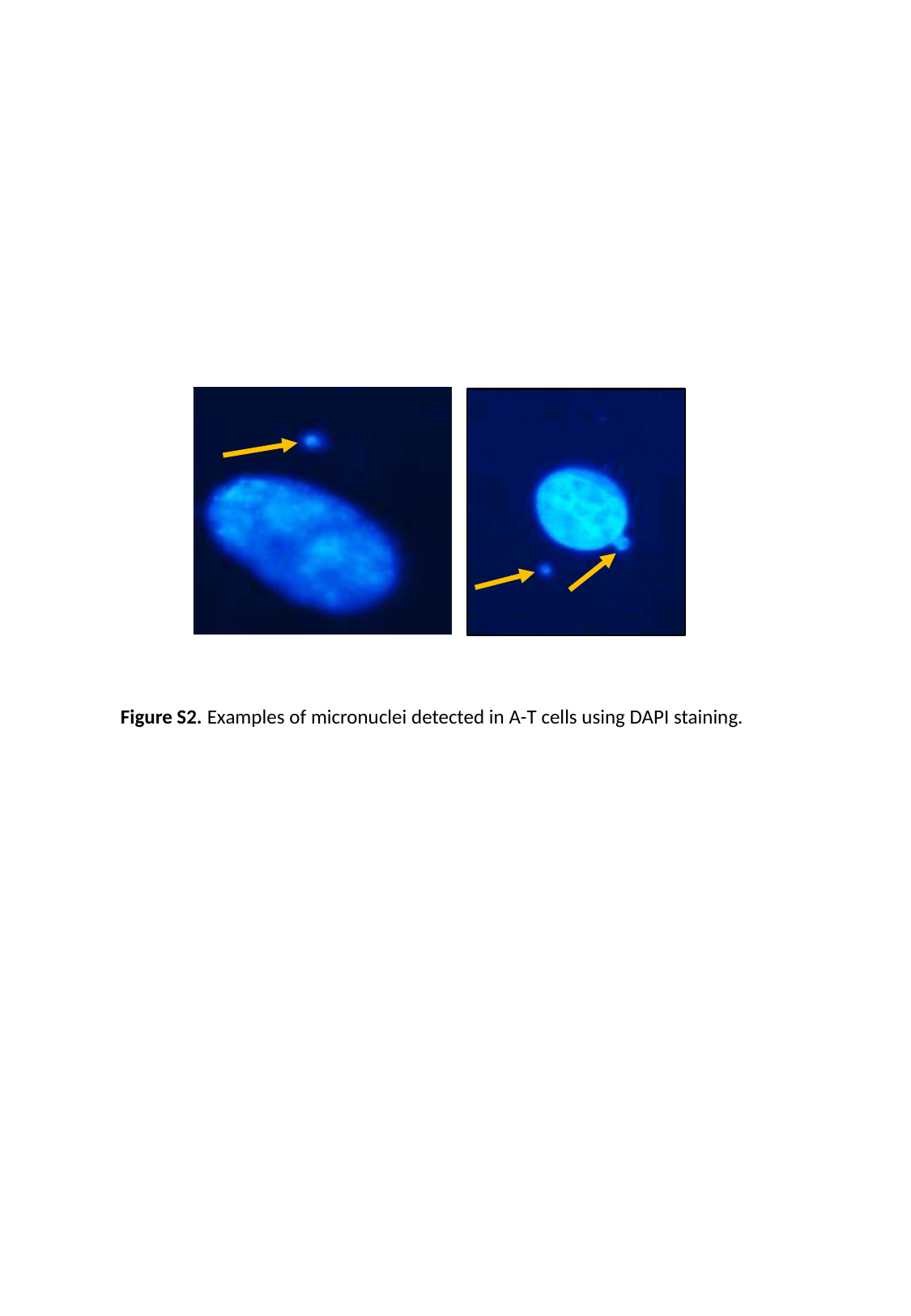

Figure S2. Examples of micronuclei detected in A-T cells using DAPI staining.

## Slide 3
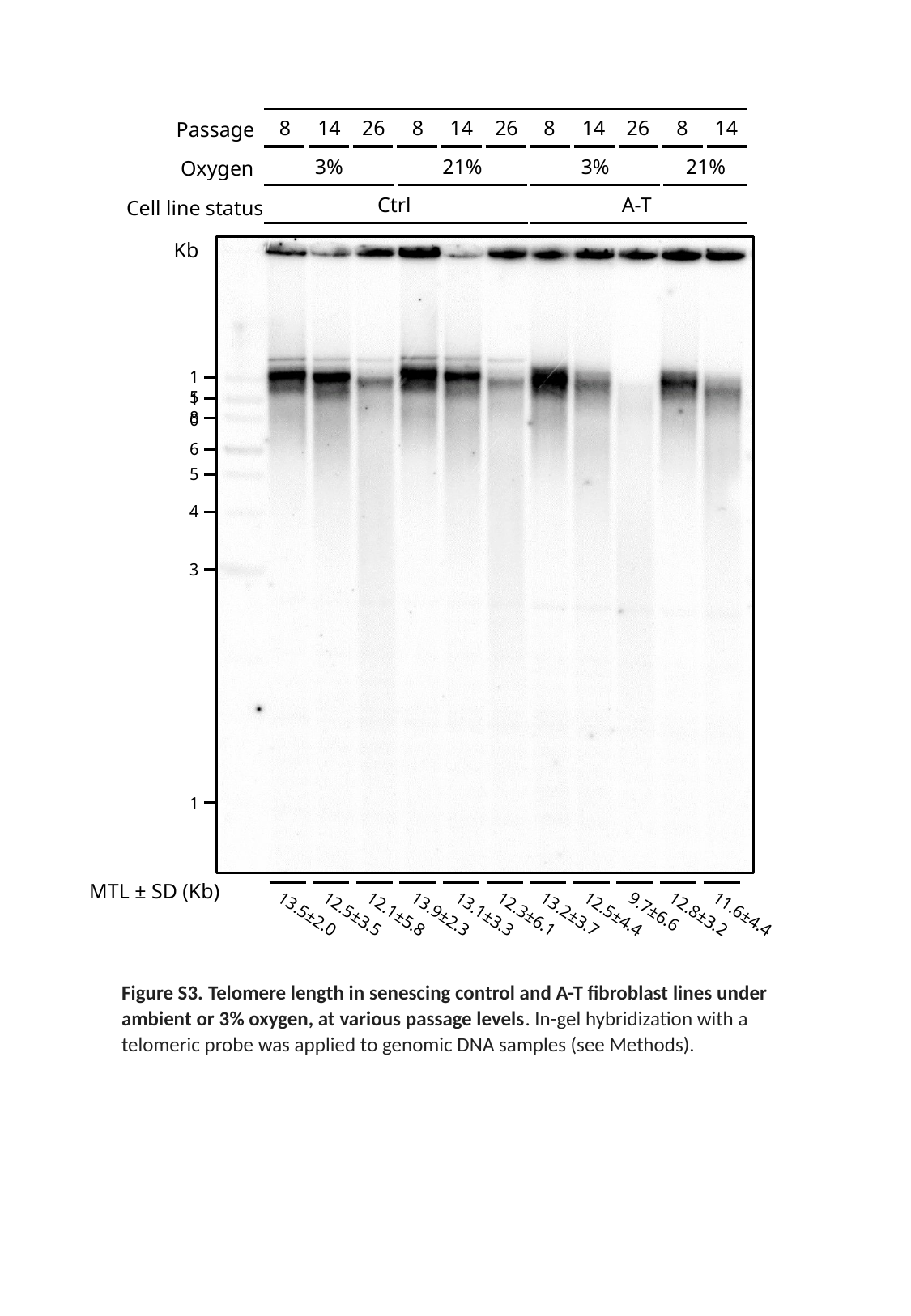

8
14
26
8
14
26
8
14
26
8
14
Passage
3%
21%
3%
21%
Oxygen
Ctrl
A-T
Cell line status
Kb
15
10
8
6
5
4
3
1
MTL ± SD (Kb)
9.7±6.6
13.5±2.0
12.5±3.5
12.1±5.8
13.9±2.3
13.1±3.3
12.3±6.1
13.2±3.7
12.5±4.4
12.8±3.2
11.6±4.4
Figure S3. Telomere length in senescing control and A-T fibroblast lines under ambient or 3% oxygen, at various passage levels. In-gel hybridization with a telomeric probe was applied to genomic DNA samples (see Methods).

## Slide 4
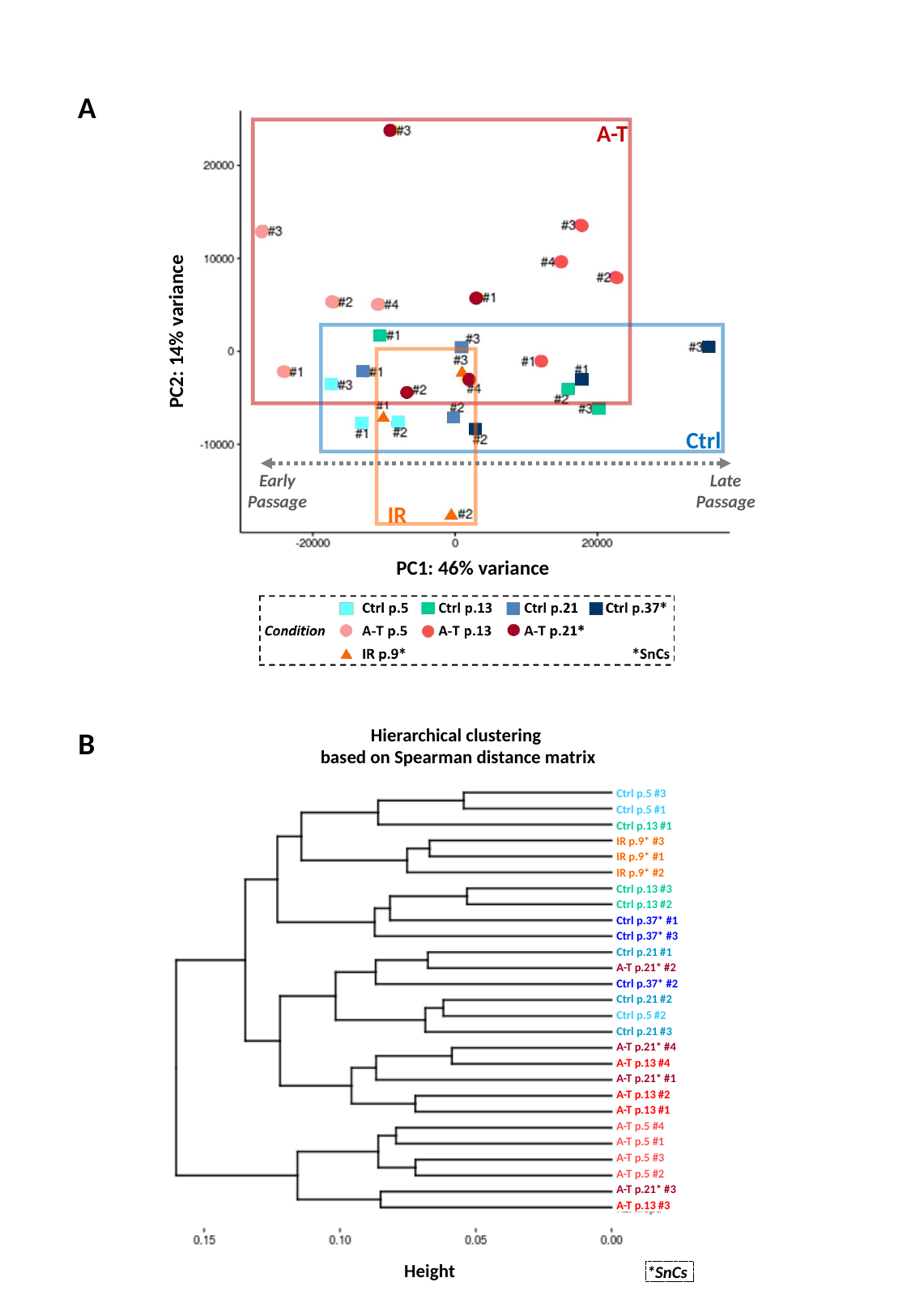

A
A-T
PC2: 14% variance
Ctrl
Early
Passage
Late
Passage
IR
PC1: 46% variance
Hierarchical clustering
based on Spearman distance matrix
Ctrl p.5 #3
Ctrl p.5 #1
Ctrl p.13 #1
IR p.9* #3
IR p.9* #1
IR p.9* #2
Ctrl p.13 #3
Ctrl p.13 #2
Ctrl p.37* #1
Ctrl p.37* #3
Ctrl p.21 #1
A-T p.21* #2
Ctrl p.37* #2
Ctrl p.21 #2
Ctrl p.5 #2
Ctrl p.21 #3
A-T p.21* #4
A-T p.13 #4
A-T p.21* #1
A-T p.13 #2
A-T p.13 #1
A-T p.5 #4
A-T p.5 #1
A-T p.5 #3
A-T p.5 #2
A-T p.21* #3
A-T p.13 #3
Height
*SnCs
B

## Slide 5
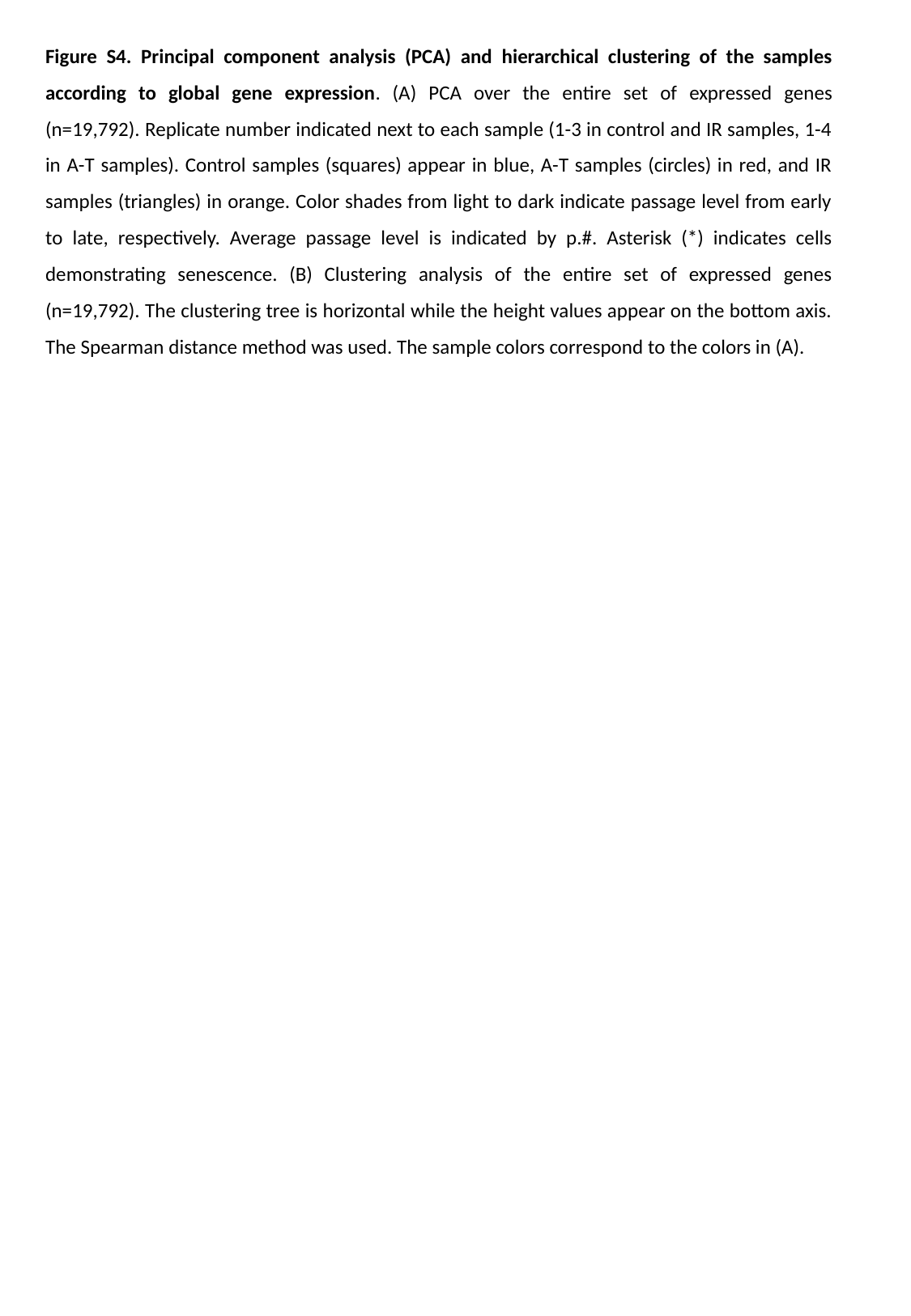

Figure S4. Principal component analysis (PCA) and hierarchical clustering of the samples according to global gene expression. (A) PCA over the entire set of expressed genes (n=19,792). Replicate number indicated next to each sample (1-3 in control and IR samples, 1-4 in A-T samples). Control samples (squares) appear in blue, A-T samples (circles) in red, and IR samples (triangles) in orange. Color shades from light to dark indicate passage level from early to late, respectively. Average passage level is indicated by p.#. Asterisk (*) indicates cells demonstrating senescence. (B) Clustering analysis of the entire set of expressed genes (n=19,792). The clustering tree is horizontal while the height values appear on the bottom axis. The Spearman distance method was used. The sample colors correspond to the colors in (A).

## Slide 6
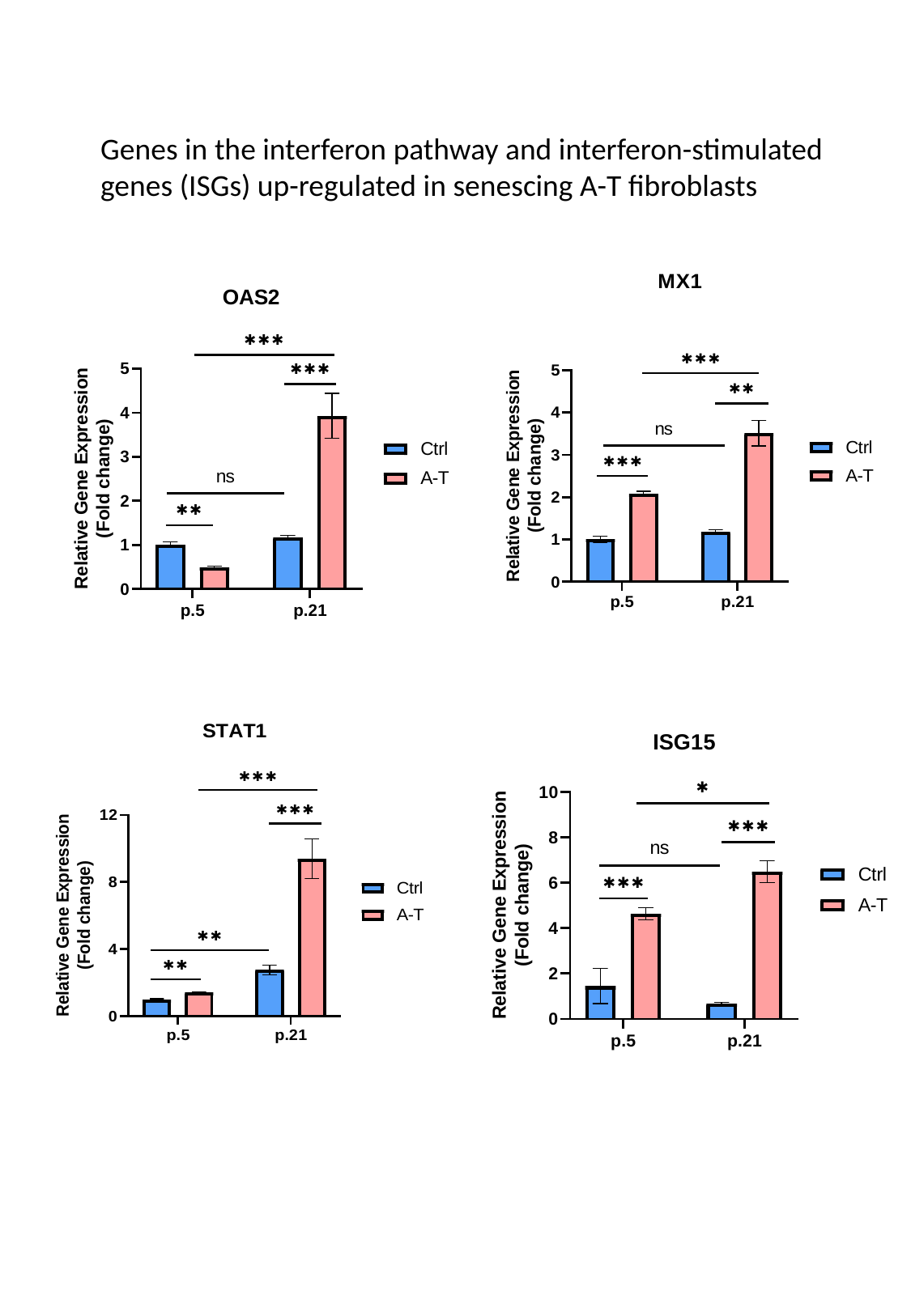

Genes in the interferon pathway and interferon-stimulated genes (ISGs) up-regulated in senescing A-T fibroblasts

## Slide 7
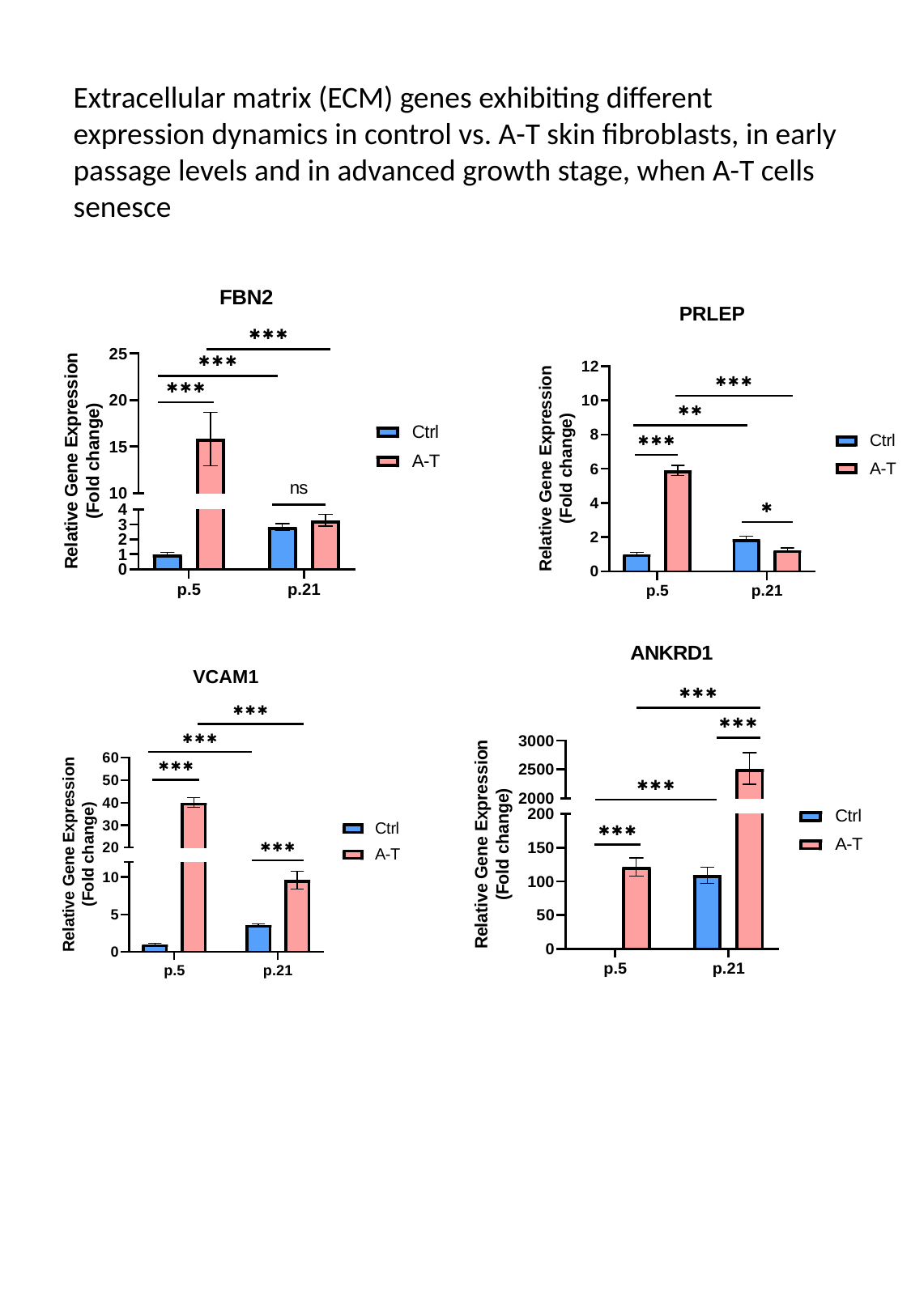

Extracellular matrix (ECM) genes exhibiting different expression dynamics in control vs. A-T skin fibroblasts, in early passage levels and in advanced growth stage, when A-T cells senesce

## Slide 8
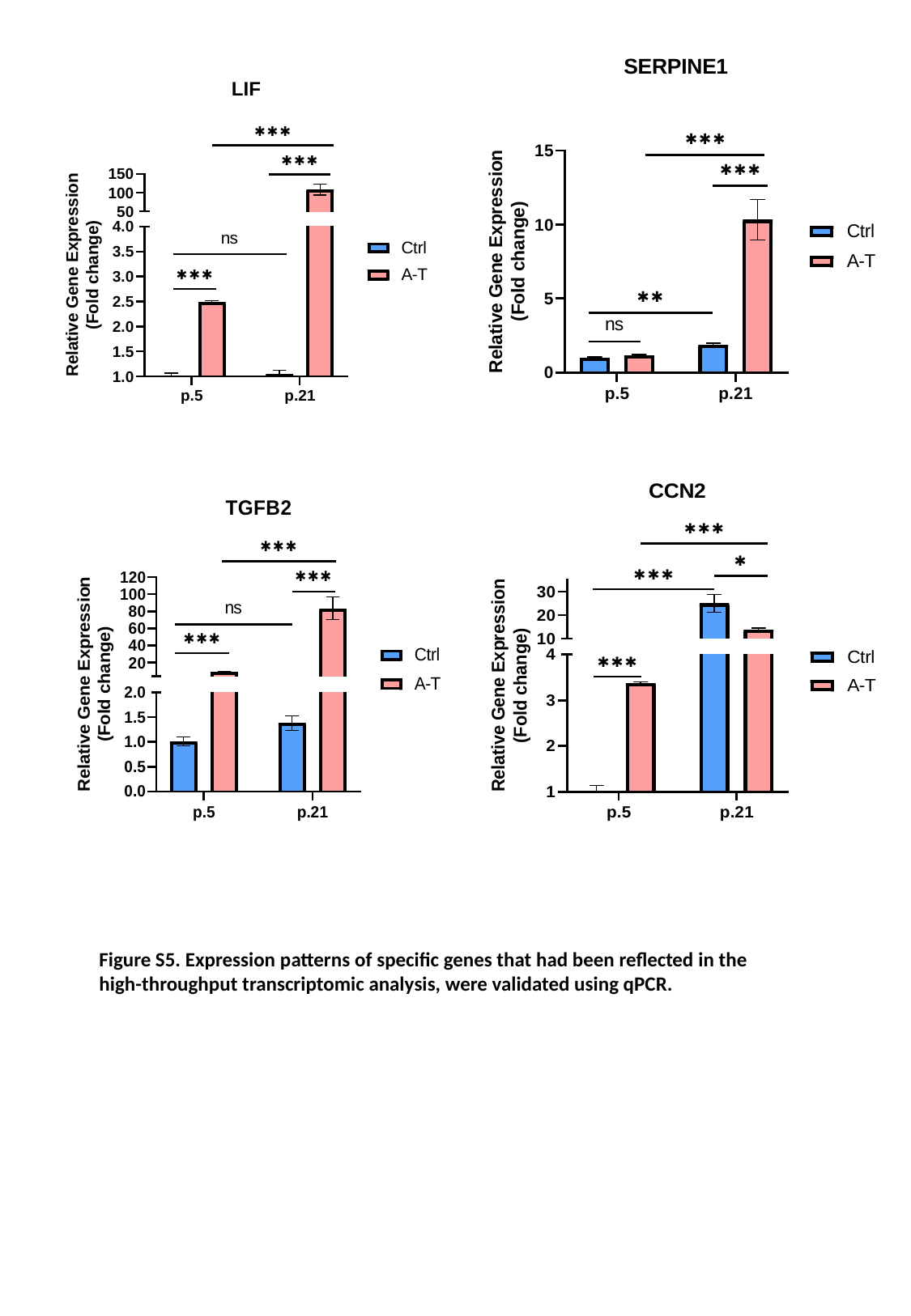

Figure S5. Expression patterns of specific genes that had been reflected in the high-throughput transcriptomic analysis, were validated using qPCR.

## Slide 9
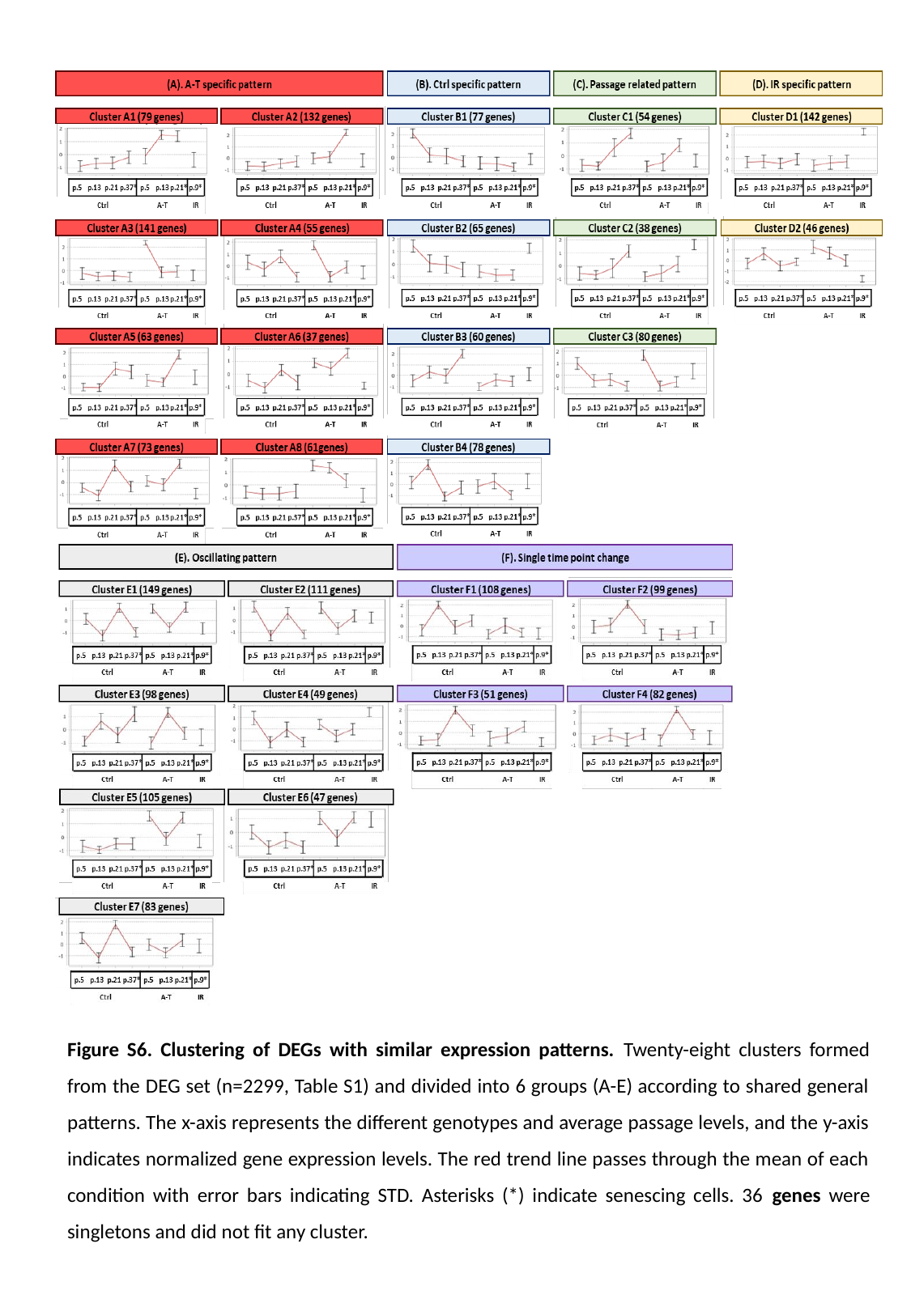

Figure S6. Clustering of DEGs with similar expression patterns. Twenty-eight clusters formed from the DEG set (n=2299, Table S1) and divided into 6 groups (A-E) according to shared general patterns. The x-axis represents the different genotypes and average passage levels, and the y-axis indicates normalized gene expression levels. The red trend line passes through the mean of each condition with error bars indicating STD. Asterisks (*) indicate senescing cells. 36 genes were singletons and did not fit any cluster.

## Slide 10
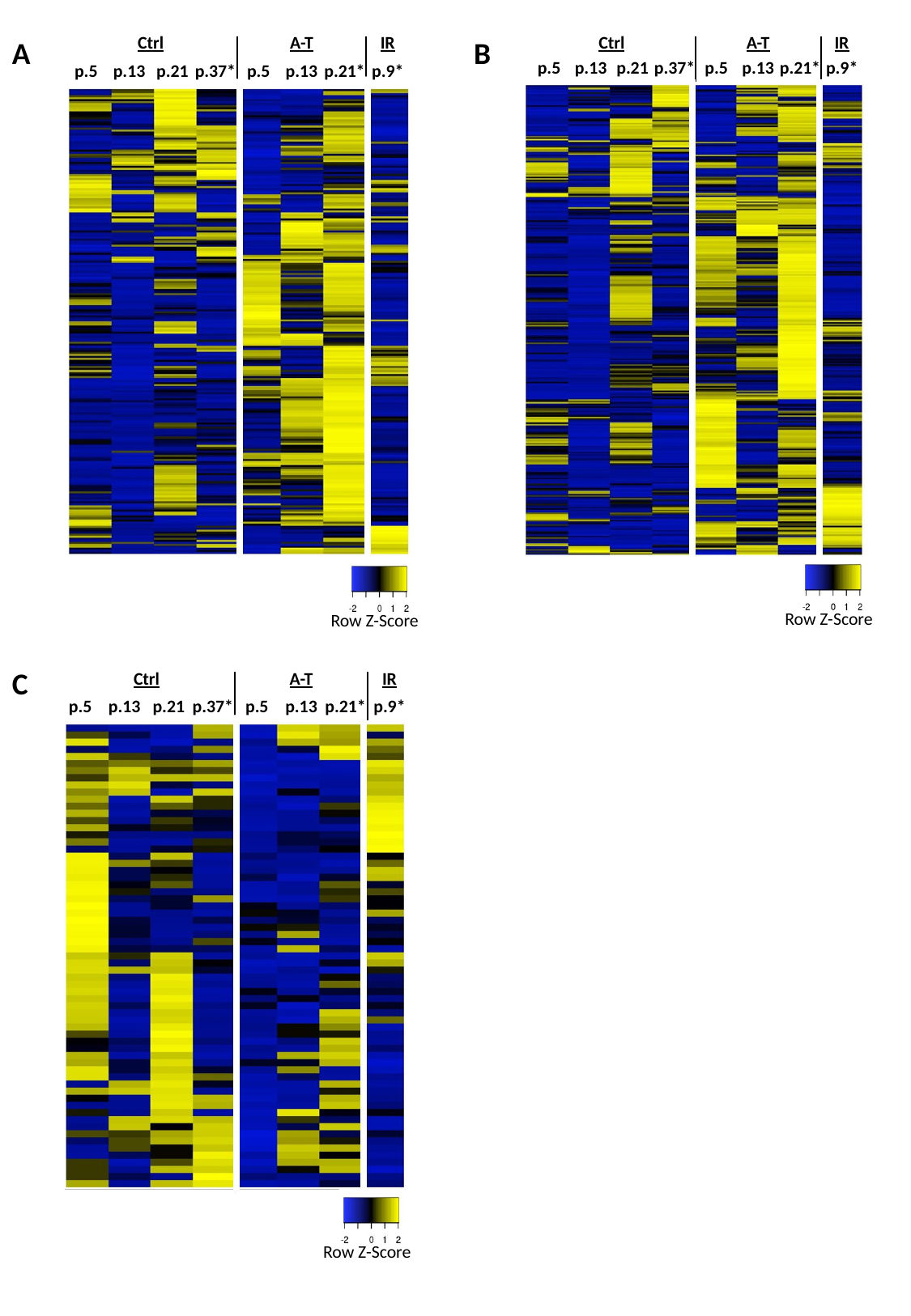

A
B
| Ctrl | | | | A-T | | | IR |
| --- | --- | --- | --- | --- | --- | --- | --- |
| p.5 | p.13 | p.21 | p.37\* | p.5 | p.13 | p.21\* | p.9\* |
| Ctrl | | | | A-T | | | IR |
| --- | --- | --- | --- | --- | --- | --- | --- |
| p.5 | p.13 | p.21 | p.37\* | p.5 | p.13 | p.21\* | p.9\* |
Row Z-Score
Row Z-Score
C
| Ctrl | | | | A-T | | | IR |
| --- | --- | --- | --- | --- | --- | --- | --- |
| p.5 | p.13 | p.21 | p.37\* | p.5 | p.13 | p.21\* | p.9\* |
Row Z-Score

## Slide 11
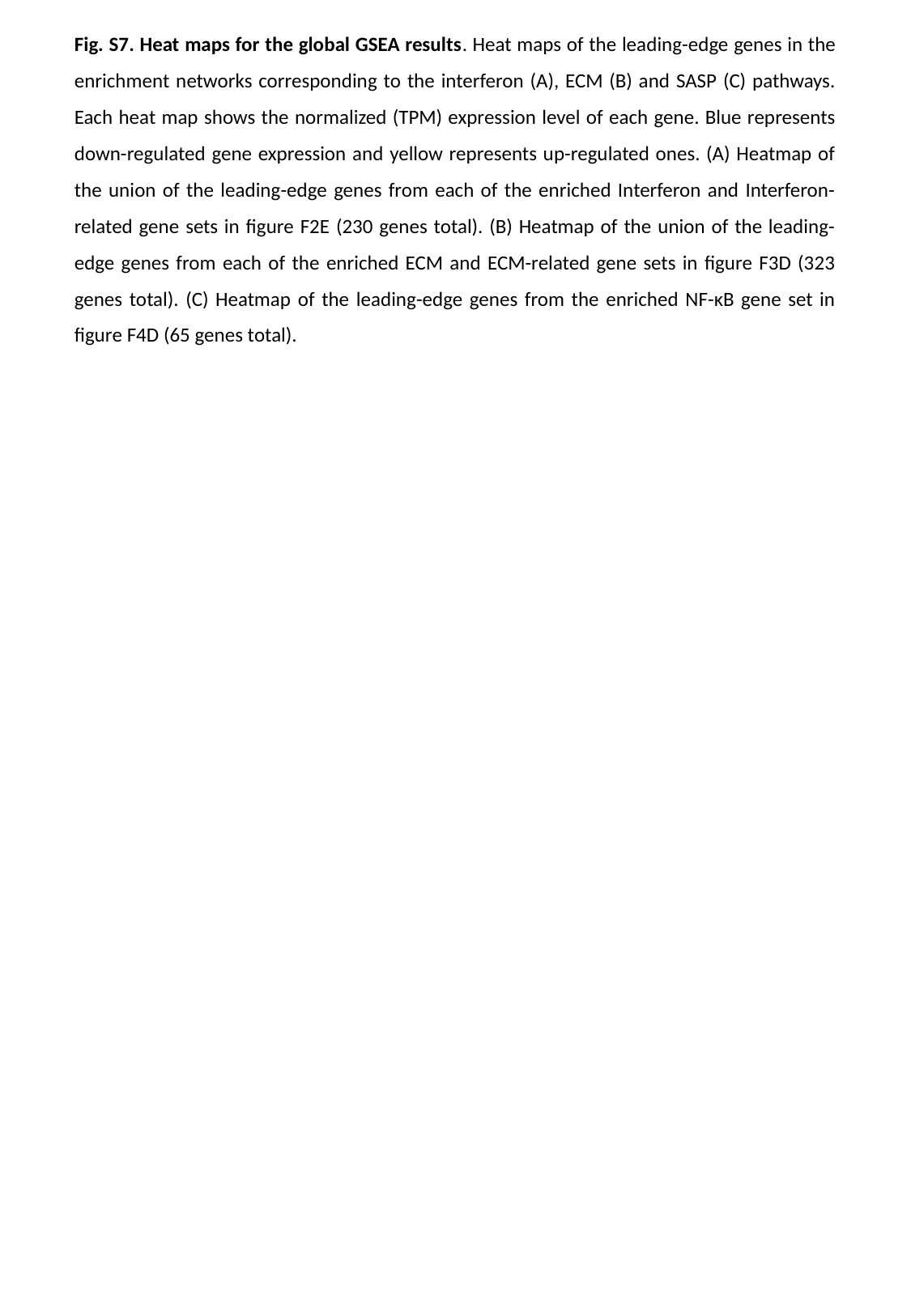

Fig. S7. Heat maps for the global GSEA results. Heat maps of the leading-edge genes in the enrichment networks corresponding to the interferon (A), ECM (B) and SASP (C) pathways. Each heat map shows the normalized (TPM) expression level of each gene. Blue represents down-regulated gene expression and yellow represents up-regulated ones. (A) Heatmap of the union of the leading-edge genes from each of the enriched Interferon and Interferon-related gene sets in figure F2E (230 genes total). (B) Heatmap of the union of the leading-edge genes from each of the enriched ECM and ECM-related gene sets in figure F3D (323 genes total). (C) Heatmap of the leading-edge genes from the enriched NF-κB gene set in figure F4D (65 genes total).

## Slide 12
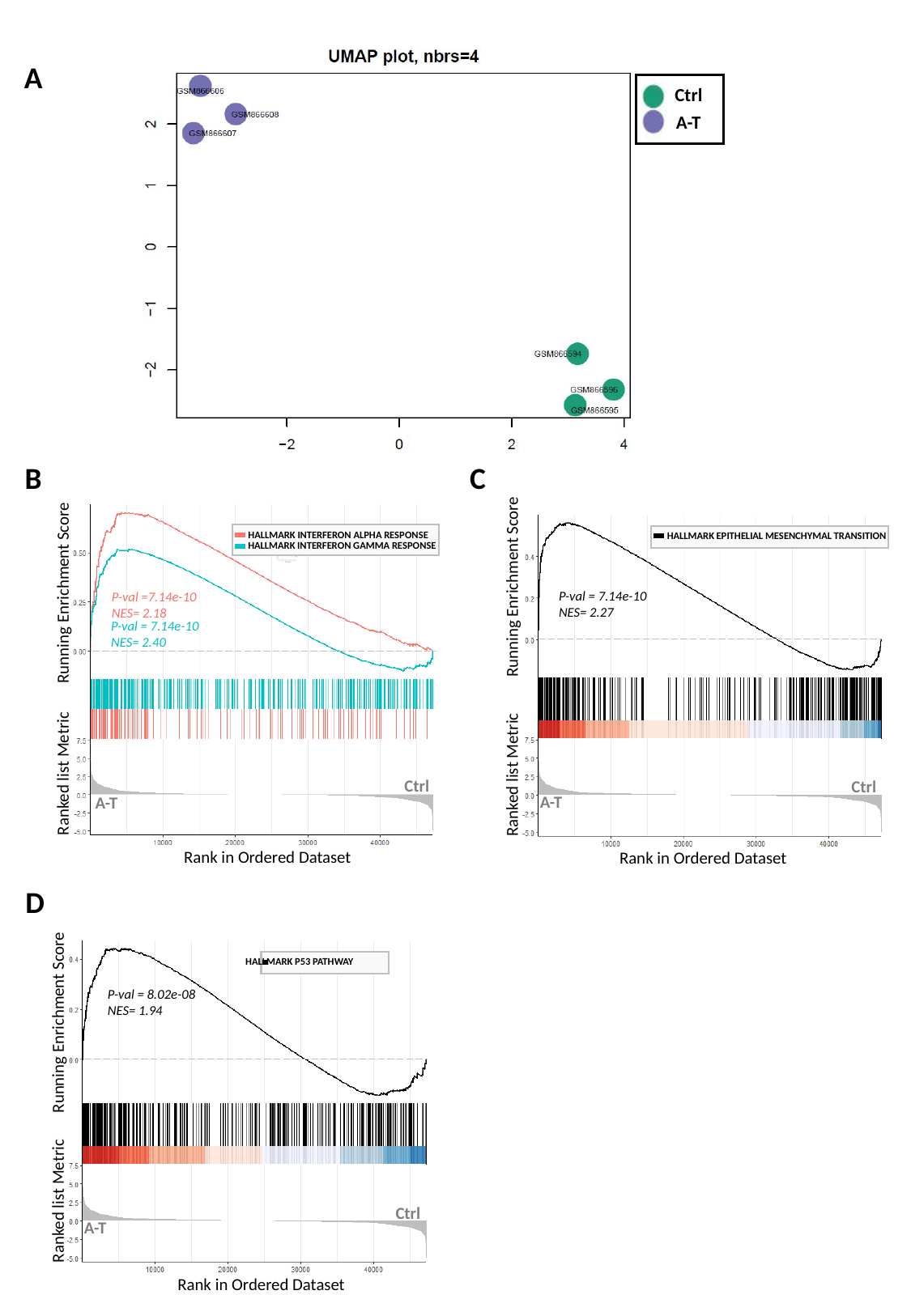

Ctrl
A-T
A
B
C
HALLMARK EPITHELIAL MESENCHYMAL TRANSITION
Running Enrichment Score
P-val = 7.14e-10
NES= 2.27
Ranked list Metric
Ctrl
A-T
Rank in Ordered Dataset
P-val =7.14e-10
NES= 2.18
P-val = 7.14e-10
NES= 2.40
HALLMARK INTERFERON ALPHA RESPONSE
HALLMARK INTERFERON GAMMA RESPONSE
Running Enrichment Score
Ranked list Metric
Ctrl
A-T
Rank in Ordered Dataset
D
HALLMARK P53 PATHWAY
P-val = 8.02e-08
NES= 1.94
Running Enrichment Score
Ranked list Metric
Ctrl
A-T
Rank in Ordered Dataset

## Slide 13
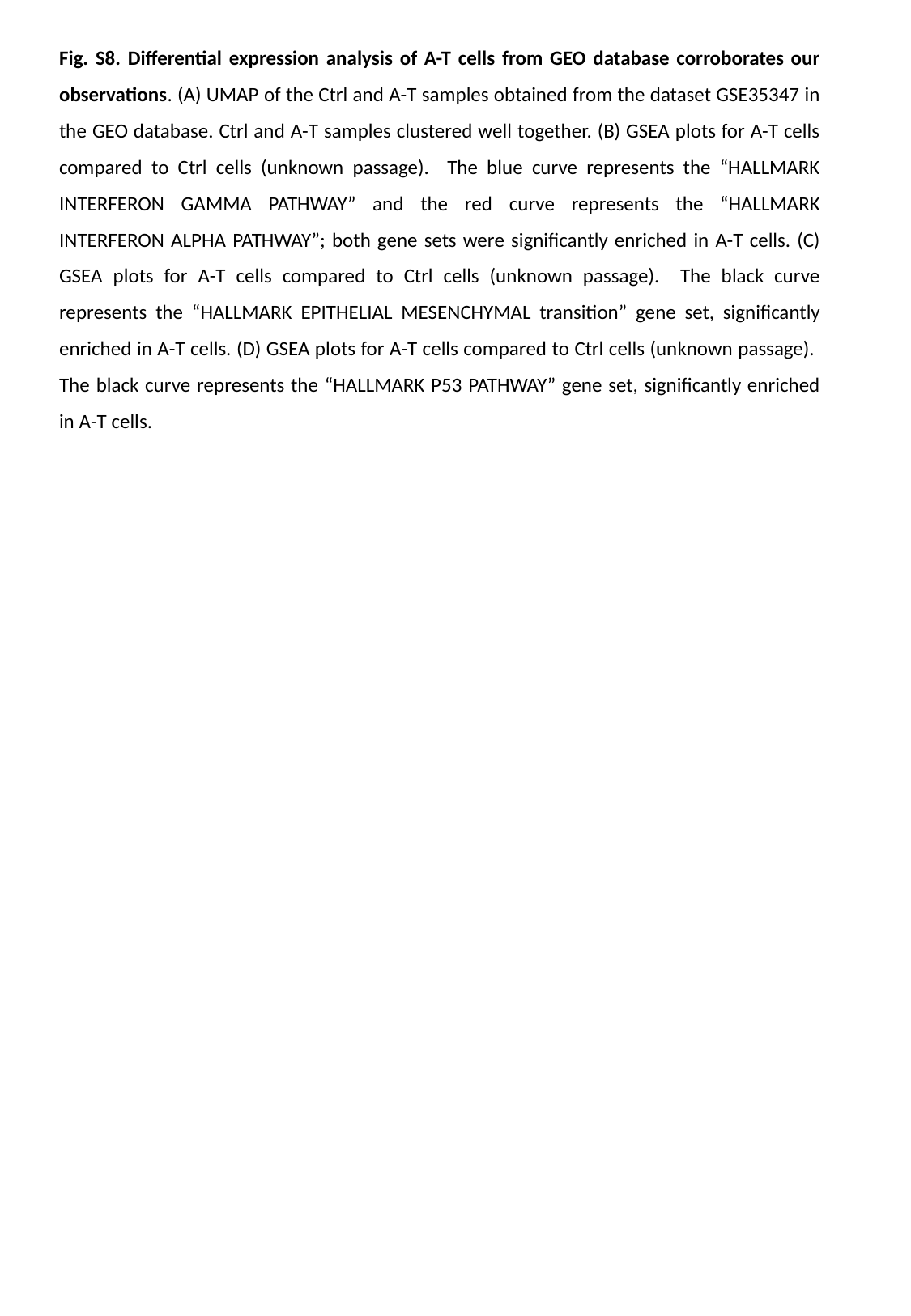

Fig. S8. Differential expression analysis of A-T cells from GEO database corroborates our observations. (A) UMAP of the Ctrl and A-T samples obtained from the dataset GSE35347 in the GEO database. Ctrl and A-T samples clustered well together. (B) GSEA plots for A-T cells compared to Ctrl cells (unknown passage). The blue curve represents the “HALLMARK INTERFERON GAMMA PATHWAY” and the red curve represents the “HALLMARK INTERFERON ALPHA PATHWAY”; both gene sets were significantly enriched in A-T cells. (C) GSEA plots for A-T cells compared to Ctrl cells (unknown passage). The black curve represents the “HALLMARK EPITHELIAL MESENCHYMAL transition” gene set, significantly enriched in A-T cells. (D) GSEA plots for A-T cells compared to Ctrl cells (unknown passage). The black curve represents the “HALLMARK P53 PATHWAY” gene set, significantly enriched in A-T cells.
