# Supplementary material for: Accelerated replicative senescence of ataxia‐telangiectasia skin fibroblasts is retained at physiologic oxygen levels, with unique and common transcriptional patterns
Source: Aging Cell. 2023 May 30;22(8):e13869. doi: 10.1111/acel.13869 (PMC10410012; doi:10.1111/acel.13869)
Supplement: Supplementary file 2 — Table S1. Table S2. Table S3. [file ACEL-22-e13869-s002.docx]

**Table S1. Cell lines used in this study**

| Cell lines used for assessing growth and senescence parameters | | |
| --- | --- | --- |
| Annotation | **Genotype** | ***ATM* mutations** |
| F-89 | *ATM+/+* |  |
| F-2056 | *ATM+/+* |  |
| F-2053 | *ATM+/+* |  |
| 2111 | *ATM-/-* | Homozygous for 5653delA (frameshift leading to protein truncation) |
| 2090 | *ATM-/-* | Unknown |
| 2016* | *ATM-/-* | Homozygous for c.T103C  (p.R35X, protein truncation) |
| Cell lines used for RNA-Seq | | |
| Cell line annotation | **Genotype** | ***ATM* mutation** |
| F-2021 | *ATM+/+* |  |
| F-2056 | *ATM+/+* |  |
| F-2068 | *ATM+/+* |  |
| F-2116 | *ATM-/-* | Compound heterozygote. Mutation 1: c. del1027-1030 (frameshift, protein truncation). Mutation 2: c.1065+1 G>T (donor splice site abolished; protein truncation). |
| F-2111 | *ATM-/-* | Homozygous for 5653delA (protein truncation) |
| F-2115 | *ATM-/-* | Unknown |
| F-2065* | *ATM-/-* | Homozygous for c.T103C  (p.R35X, protein truncation) |

*Moroccan-Jewish descent. The c.T103C *ATM* mutation is a founder mutation in this community.

**
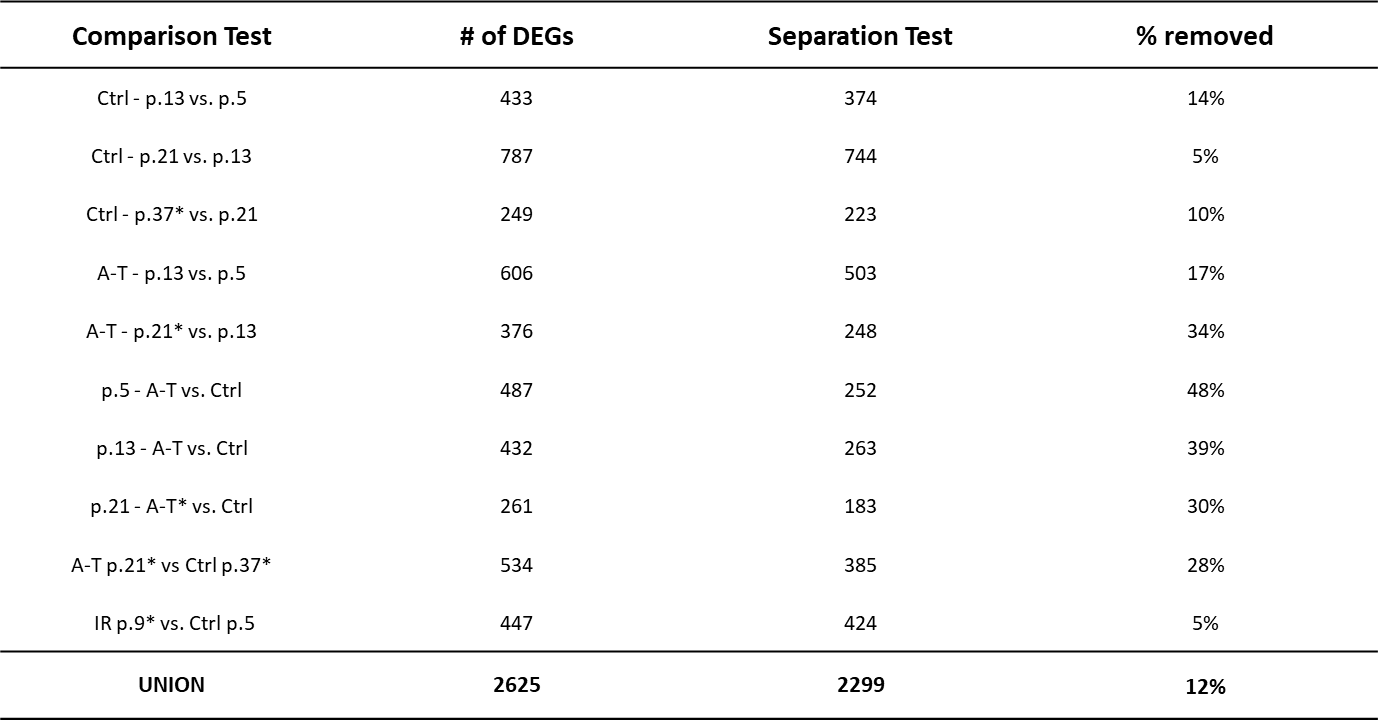
**

**Table S2. Number of genes passing the differential expression and separation tests thresholds**. The first column on the left shows the comparison test performed. The p.# is the average passage level. The second column presents the numbers of differentially expressed genes (DEGs), defined as genes with fold-change >1.5 or <-1.5, passing adjusted p-value <0.05. The following column indicates how many of these genes passed separation tests (see Materials and Methods), and the last column shows the fraction of genes removed on account of high separation. The bottom row indicates the union results. 2299 genes passed both tests.

**
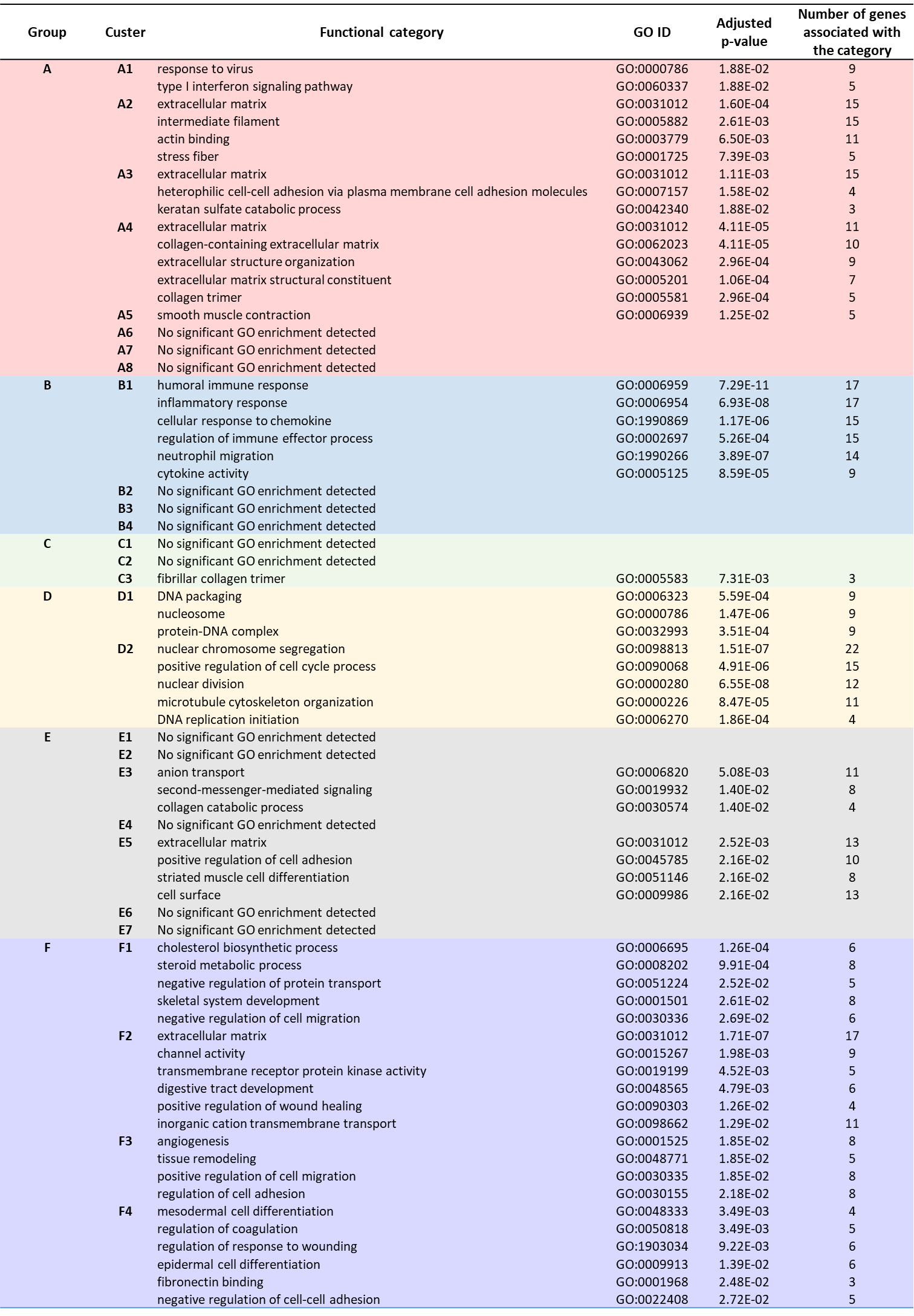
**

**Table S3. Functional GO enrichment in the gene clusters.** Major GO functional groups in clusters shown in Figure SF4. A significant enrichment of a functional group required p<0.05.
